# Electrospun Conducting Polymers: Approaches and Applications

**DOI:** 10.3390/ma15248820

**Published:** 2022-12-09

**Authors:** Mariana Acosta, Marvin D. Santiago, Jennifer A. Irvin

**Affiliations:** 1 Materials Science, Engineering and Commercialization Program, Texas State University, San Marcos, TX 78666, USA; 2 Department of Chemistry and Biochemistry, Texas State University, San Marcos, TX 78666, USA

**Keywords:** conducting polymers, electrospinning, nanocomposite, nanofibers

## Abstract

Inherently conductive polymers (CPs) can generally be switched between two or more stable oxidation states, giving rise to changes in properties including conductivity, color, and volume. The ability to prepare CP nanofibers could lead to applications including water purification, sensors, separations, nerve regeneration, wound healing, wearable electronic devices, and flexible energy storage. Electrospinning is a relatively inexpensive, simple process that is used to produce polymer nanofibers from solution. The nanofibers have many desirable qualities including high surface area per unit mass, high porosity, and low weight. Unfortunately, the low molecular weight and rigid rod nature of most CPs cannot yield enough chain entanglement for electrospinning, instead yielding polymer nanoparticles via an electrospraying process. Common workarounds include co-extruding with an insulating carrier polymer, coaxial electrospinning, and coating insulating electrospun polymer nanofibers with CPs. This review explores the benefits and drawbacks of these methods, as well as the use of these materials in sensing, biomedical, electronic, separation, purification, and energy conversion and storage applications.

## 1. Introduction

**Figure 1:**
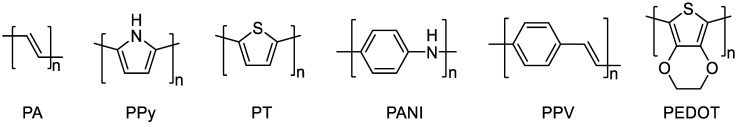
Chemical structures of the repeat units of some representative CPs; from left to right: polyacetylene (PA), polypyrrole (PPy), polythiophene (PT), polyaniline (PANI), poly(phenylene vinylene) (PPV), and poly(3,4-ethylenedioxythiophene (PEDOT).

Inherently conductive polymers (CPs) possess conjugated backbones that can be reversibly oxidized and reduced via chemical or electrochemical means. Changes in oxidation state are accompanied by changes in properties including conductivity, color, reactivity, and volume. In the year 2000, the Nobel Prize in Chemistry was awarded to Alan Heeger, Alan MacDiarmid, and Hideki Shirakawa, for their critical work in the field of CPs. CPs have superior electrical and optical properties that are comparable with those of metals and inorganic semiconductors [1]. They display high conductivity/weight ratios and excellent control of the electrical stimulus [2]. CPs are used in a wide range of organic electronic applications including organic photovoltaic cells [1,3,4,5,6,7,8], organic light-emitting diodes (OLEDs) [1,3,7,8,9,10,11], field-effect transistors (FETs) [3,12,13,14], organic thin-film transistors (OTFTs) [7,15,16,17], electrochromic devices [3,4,18,19,20,21,22], antistatic/anticorrosion coatings [1,7,23,24,25,26,27,28], batteries [1,29,30,31], and chemical sensors [32,33,34]. The chemical, electrical, and physical properties of CPs can also be specifically tailored to the needs of the application by incorporating antibodies, enzymes, or other biological moieties [2,35,36,37,38,39,40,41,42,43,44,45]. Various CPs, including polyaniline (PANI), polypyrrole (PPy), and polythiophene (PTh) (Figure 1) have demonstrated biocompatibility, redox stability, conductivity, and excellent electrical and optical properties [1,46,47,48]. Even after synthesis, some of the properties of these CPs can be altered and controlled through stimulation (e.g., changes in electricity, pH, and light) [2,40,41,49] indicating that CPs can be used as actuators [7,43]. This makes these CPs excellent candidates for applications in biomedicine, tissue/nerve engineering applications [50,51], biosensors [44,45], and bioelectronic devices [7,52]. CP nanofibers would be particularly useful for electroactive fabrics, sensors, actuators, and tissue engineering.

Electrospinning is a versatile and straightforward way of producing nanofibers from a variety of materials, including polymers, composites, semiconductors, and ceramics [53]. Although electrospinning was first developed in the early 1900s, it did not attain widespread implementation until the 1990s [54]. Heightened interest in nanofibers and their potential applications led scientists and engineers to rediscover electrospinning, generally fabricating their own equipment from publicly available schematics to meet their individual experimental needs [55,56,57,58].

While many researchers still prefer this route, for both economic feasibility and ease of customization, the demand for production-scale electrospinning equipment is also growing. Several companies worldwide now specialize in manufacturing, selling, and servicing electrospinning equipment [59,60]. These commercial electrospinning machines can produce roll to roll quantities of nanofibers of about 200 g/h, a 20-fold increase in rate over the 100 mg/h produced with lab-scale equipment [61,62].

Nanofibers produced via electrospinning can be manipulated in a variety of different ways, and their final diameter, alignment, and structure all depend on the parameters used during the electrospinning process. For example, different electrospinning setups can produce nanofibers ranging in diameter from 1.6 nm to 2000 nm [63,64,65]. The versatility of electrospun nanofibers enables them to be applied in many different contexts. In biomedicine, nanofibers are used in blood vessel/tissue engineering [64,66,67,68], artificial nerves/muscles [65,66,67,68,69,70,71,72], biomedical scaffolds [70,73,74,75], drug delivery [68,76,77,78], and wound healing [72,76,79,80]. Outside of the biomedical field, applications include electrochemical sensors [72,81,82], high-performance filtration [65,72,83,84,85], energy storage [86,87,88], smart textiles [89,90,91,92,93], and photonic/electronic devices [94,95,96]. 

**Figure 2:**
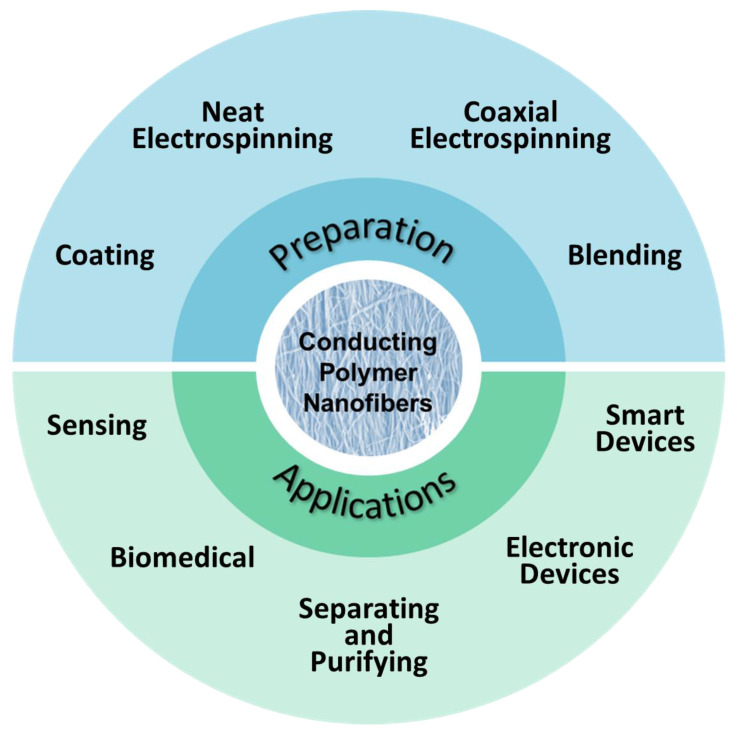
This review article summarizes methods of preparation of CP nanofibers and their application in several areas.

A wide variety of inexpensive, commercially available (“commodity”) polymers have been electrospun, but there are inherent difficulties associated with electrospinning CPs. This review focuses on the production of CP nanofibers via electrospinning, as well as the applications of CP nanofibers (Figure 2). A brief background on CPs and the electrospinning process is provided, followed by a discussion of the approaches used to incorporate CPs into electrospun nanofibers. A summary of recent advances and suggestions for future work in electrospun CP applications conclude this review. Many thousands of publications on electrospun CP nanofibers are available; thus, this review focuses on some exemplary highlights of efforts in this area.

## 2. Inherently Conducting Polymers

**Figure 3:**
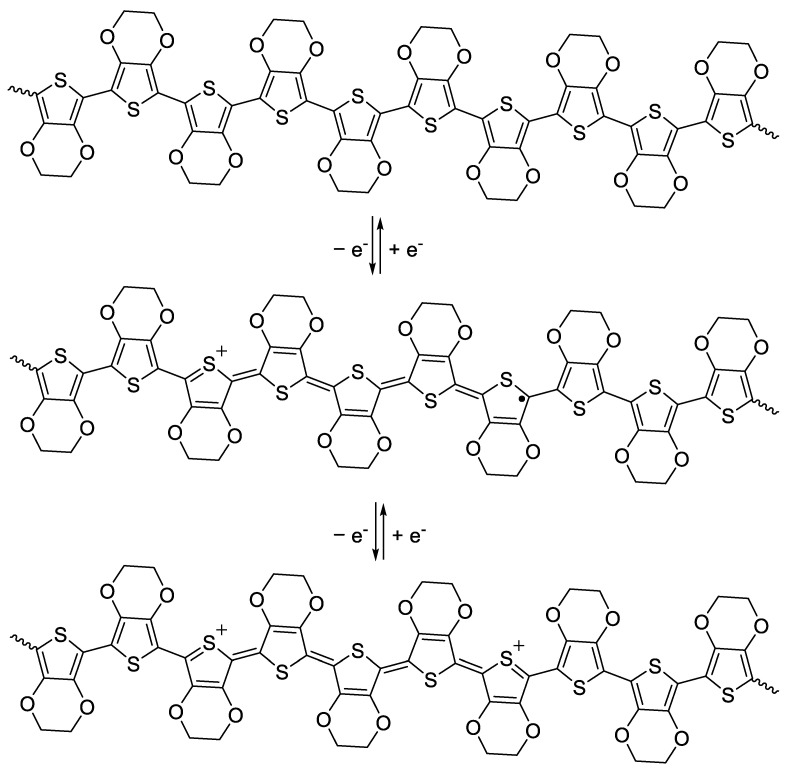
Reversible oxidation and reduction processes form resonance-delocalized electrons and cations (holes) along the backbone of CPs such as PEDOT. Redox processes, which are typically reversible, allow interconversion among neutral polymers (**top**), radical cations known as polarons (**middle**), and dications known as bipolarons (**bottom**). Reprinted from Ref. [45], available at https://www.mdpi.com/1996-1944/12/16/2629; accessed on 1 December 2022.

CPs are highly conjugated polymers containing alternating single and double bonds, as shown in Figure 1; this sp^2^ hybridized backbone is responsible for the unique electronic and optical properties of CPs. Resonance delocalization of electrons and cations (holes) formed during the oxidation (doping) process (Figure 3) allows for the conductivity of CP systems to increase from the semiconducting regime to the metallic regime when oxidized [4]. Oxidation of the neutral polymer (Figure 3, top) initially forms the polymer radical cation, known as a polaron (Figure 3, middle). Further removal of electrons forms the polymer dication, known as a bipolaron (Figure 3, bottom). As the polymer oxidation state changes, CP properties including color, solubility, conductivity, volume, and reactivity, also change [45,97]. The process is typically reversible; CPs can typically be oxidized and reduced thousands to millions of times, enabling their use in a wide range of devices [98,99]. Several excellent reviews on the synthesis, characterization, properties, and applications of CPs are available [3,7,99,100,101,102]. 

The rigid structure of most CPs makes them highly insoluble, and many are sensitive to moisture and air. They are also difficult to process and characterize; neat polyacetylene, albeit highly conductive, has been shown to be poorly suited for any technological applications [103,104,105].

Substituent groups can be incorporated into CPs to improve solubility and stability [28,29], control the band gap (E_g_) [22], and decrease the oxidation potential [106,107,108] of the monomers and their resulting polymers. The stability, conductivity, and processability of these CP derivatives can also be improved through careful structural modification [109,110,111]. 

**Figure 4:**
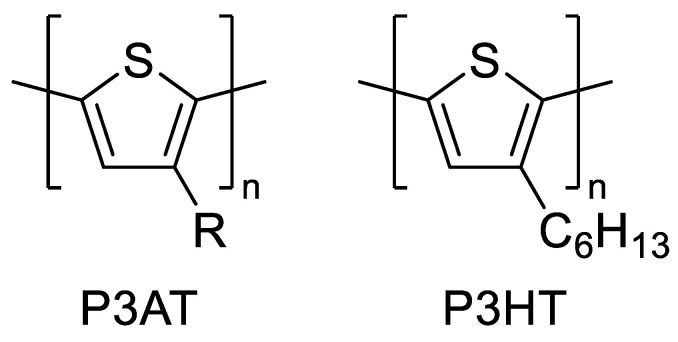
**Left**: General structure of poly(3-alkylthiophenes) (P3AT); **Right**: poly(3-hexylthiophene) (P3HT).

Polythiophenes have become one of the most widely used and researched CPs primarily because of their relative stability in oxygen and moisture [112]. While polythiophene (PT) is an insoluble material, polymerization of alkyl-substituted thiophenes with propyl or longer alkyl chains yields poly(3-alkylthiophene)s (P3ATs, Figure 4) that are soluble in organic solvents and mechanically processable. Long-chain alkoxy groups exhibit several advantages over alkyl substituents, such as increased solubility and a significant reduction in polymer E_g_ due to the electron-donating nature of the alkoxy substituent [109,110,111]. Poly(3-hexylthiophene) (P3HT) (Figure 4) is one of the most notable and widely researched CPs. 

**Figure 5:**
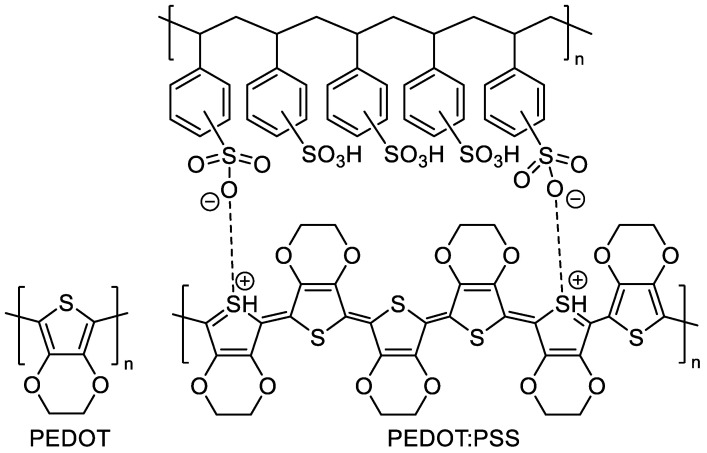
**Left***:* Structure of poly(3,4-ethylenedioxythiophene (PEDOT); **Right**: structure of PEDOT:PSS.

Incorporation of ether substituents to conjugated polyheterocycles further increases their electron density and reduces their oxidation potential [106]. To minimize steric interactions, researchers have focused primarily on poly(3,4-ethylenedioxythiophene) (PEDOT) and its derivatives (Figure 5). PEDOT is an insoluble conjugated polymer that can be functionalized via incorporation of alkyl and/or alkoxy substituents to impart solubility [113,114,115,116]. Doping PEDOT with poly(styrene sulfonate) yields stable, water-dispersible nanoparticles of poly(3,4-ethylenedioxythiophene):poly(styrene sulfonate) (PEDOT:PSS, Figure 5) that can be used as an electrostatic dissipation coating for photographic film [117]. PEDOT:PSS is now one of the most widely commercially used soluble conductive polymers in the world, with over 100 tons produced annually [7].

## 3. Electrospinning

### 3.1. Electrospinning Fundamentals

The history of electrospinning and the development of electrospinning design assemblies have been thoroughly reviewed by Tucker et al. [118], Teo and Ramakrishna [53], and Ghosal et al. [119]. For a more complete foundation on electrospinning and the future of electrospinning, we recommend studies by Ramakrishna et al. [59,120], Baji et al. [121], Haider et al. [122], and Xue et al. [72]. A brief discussion of polymer and system parameters impacting electrospinning and how they pertain to CPs is covered in sections below.

**Figure 6:**
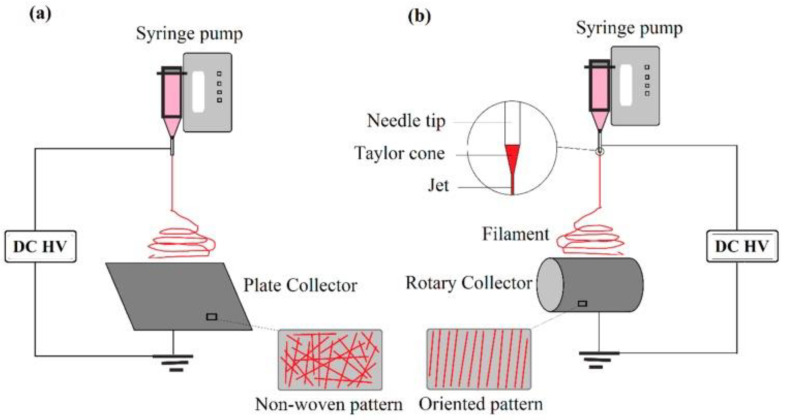
A typical electrospinning set up [130] using (**a**) a static grounded collector plate and (**b**) a grounded rotating drum collector. Reprinted from Ref. [130], available at https://doi.org/10.3390/ma10111238; accessed on 1 December 2022.

Electrospinning setups are versatile and commercially scalable, with various manufacturers making and selling industrial-sized electrospinning equipment [123,124,125,126]. In its most basic form, an electrospinning setup simply requires a high voltage power supply, a syringe pump, a syringe, a needle, and a collection plate [127]. When an electrical charge is applied, Coulombic repulsion forces are generated in the polymer solution between charges of the same polarity; these forces destabilize the hemispherical droplet of the polymer solution at the tip of the nozzle to form a droplet of conical shape called the “Taylor cone” [128]. As the applied electrostatic field strength increases, the Coulombic repulsion force eventually exceeds that of the surface tension, resulting in the ejection of an electrically charged jet of polymer solution. This charged jet travels in a straight path for a small distance before bending instability forces it into a looping path. On the path to the collector plate, the charged jet elongates while drying out or solidifying, depositing ultrafine fibers on the collector [128]. Collection plates may be replaced with drums; at speeds of ≥1000 rpm, nanofiber alignment may occur (Figure 6) [127,129,130].

### 3.2. Electrospinning Parameters

The electrospinning process is controlled by two sets of parameters: polymer parameters and process parameters [122]. These can be easily modified, enabling the electrospun nanofiber characteristics to be precisely tailored. Some mechanical properties of the nanofibers are affected by the polymer properties, while other mechanical properties are affected by the electrospinning parameters [131]. Nanofiber morphologies can be tailored to fit desired applications; this ease of tailoring is one aspect that makes electrospinning a versatile technique. By fine-tuning and precisely controlling the electrospinning parameters, electrospun nanofibers with desired properties and morphologies can be produced.

#### 3.2.1. Polymer Parameters

For electrospinning purposes, relevant polymer characteristics include molecular weight, viscosity, surface tension, polydispersity, and conductivity. These properties play a role in fiber formation and fiber diameter, reduction of bead formation, and rate of nanofiber degradation [132,133]. Studies have shown that decreasing solution concentration reduces the diameter of the electrospun fibers [53]. Effects on rheological properties including electrical conductivity, dielectric strength, and surface tension in solutions have also been observed [134]. Numerous studies have been conducted, establishing the impact of molecular weights and solution concentrations on the variety of structures that are obtained via electrospinning [134]. 

**Figure 7:**
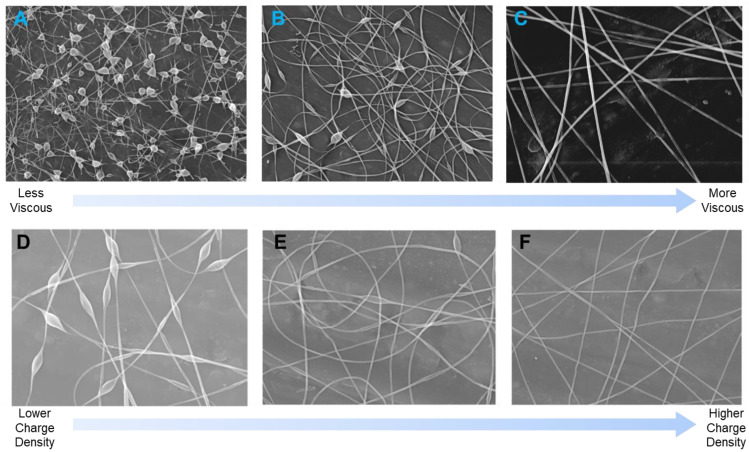
Increasing viscosity or conductivity (charge density) reduces bead formation [135]. Top: Scanning electron micrographs (SEMs) of electrospun aqueous poly(ethylene oxide (PEO). The horizontal edge of each image is 20 µm long: (**A**) 1.5 wt.% PEO, 32 cP; (**B**) 3 wt.% PEO, 289 cP; (**C**) 4 wt.% PEO, 1250 cP. Bottom: Electrospun 3 wt.% PEO with increasing NaCl content: (**D**) 15 ppm NaCl, 1.23 C·L^−1^; (**E**) 300 ppm NaCl, 3.03 C·L^−1^; (**F**) 1500 ppm NaCl, 28.8 C·L^−1^. Reprinted from Ref. [135], Copyright 1999, with permission from Elsevier.

Solution concentration/viscosity, charge density, and surface tension determine nanofiber diameter and surface morphology [135]. These parameters must be adjusted to prevent the formation of an electrospray and formation of beads during nanofiber formation [136]. Increasing solution viscosity reduces bead formation and increases fiber diameter, while increasing solution conductivity reduces bead formation and decreases fiber diameter (Figure 7) [135]. For a homogeneous solution of a linear polymer, the Huggins equation [137] (1) describes the solution viscosity as

(η_sp_/*c*) = [η] + k_H_[η]^2^*c*, (1)

where η_sp_ is the specific viscosity, [η] is the intrinsic viscosity, *c* is the polymer concentration, and k_H_ is the Huggins coefficient. Because [η] is related to the viscosity average molecular weight (M_v_) of a linear polymer by the Mark–Houwink–Sakurada equation [138], [η] is dependent on the polymer structure, solvent, and temperature. 

Polymer solution viscosity is related to the extent of polymer chain molecular entanglement within that solution. Polymer chain entanglement and, therefore, solution viscosity can be increased by increasing either the concentration or the strength of intermolecular interactions (specifically, by incorporating functional groups that are capable of dipole-dipole interactions). Viscous solutions are necessary to overcome the electrostatic and coulombic repulsion forces that stretch the electrospinning jet. At lower viscosities, the jet partially breaks up, and the free solvent molecules in the solution instead form spheres, resulting in the formation of beads [139]. 

**Figure 8:**
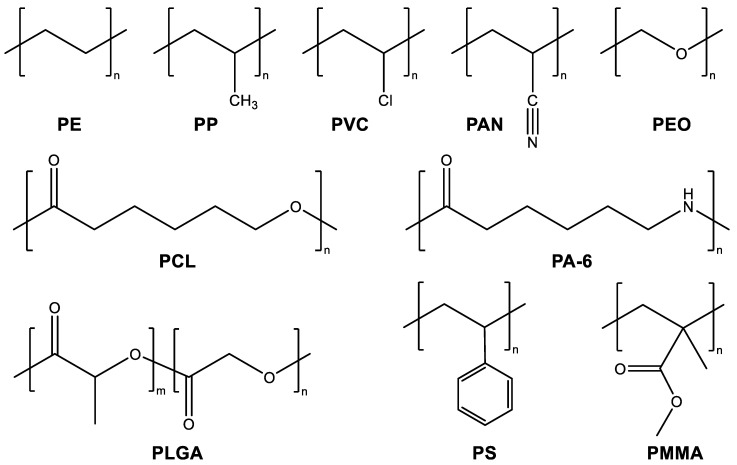
Synthetic polymers commonly used for electrospinning include: polyethylene (PE), polypropylene (PP), poly(vinyl chloride) (PVC), poly(acrylonitrile) (PAN), poly(ethylene oxide) (PEO), polycaprolactone (PCL), polycaprolactam (also known as nylon 6 and polyamide 6, PA-6), poly(lactic-*co*-glycolic acid) (PLGA), polystyrene (PS), and poly(methyl methacrylate) (PMMA).

A wide variety of organic polymers have been electrospun, including polyethylene (PE), polypropylene (PP), poly(vinyl chloride) (PVC), poly(acrylonitrile) (PAN), poly(ethylene oxide) (PEO), polycaprolactone (PCL), polycaprolactam (also known as nylon 6 and polyamide 6, PA-6), poly(lactic-*co*-glycolic acid) (PLGA), polystyrene (PS), and poly(methyl methacrylate) (PMMA) (Figure 8). Natural biopolymers, including silk fibroin, chitosan, and collagen, are also widely used in electrospinning research. When nanoscale components such as nanoparticles [140,141,142], nanotubes [143,144,145], nanowires [146,147], or nanorods [148,149,150] are incorporated into the polymer solutions, electrospun nanocomposites that combine the properties of both components can be generated. 

Simple linear polymers like PE, PP, and PS are ruled by weak van der Waals forces [151,152] and require high polymer solution concentrations to be stable enough for electrospinning. Polymers with strong intermolecular forces such as polyamides (PA-6, for example) and polyesters (PCL, for example) [153,154] exhibit increased chain entanglement, allowing lower concentrations of these polymers to be used for electrospinning. Chain entanglement is also increased in branched polymers compared to their linear polymer counterparts [155]. 

#### 3.2.2. Process Parameters

**Figure 9:**
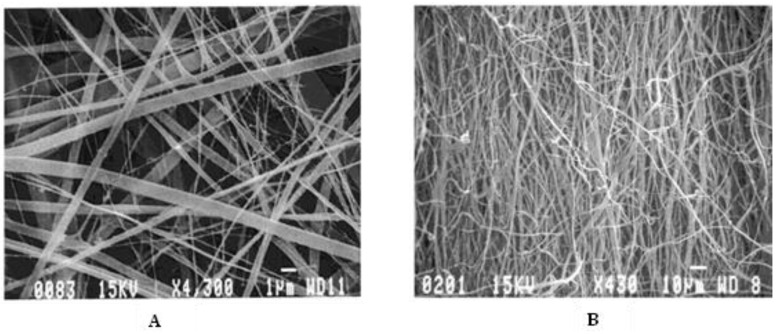
SEMs of electrospun collagen [157]. (**A**) Randomly oriented fibrils generated at less than 500 rpm. (**B**) Aligned fibrils generated at 4500 rpm. Reprinted and adapted with permission from [157]. Copyright 2002, American Chemical Society.

Process parameters, such as needle tip diameter, shape, and location, flow rate of polymer solution, electric potential, solution temperature, and relative humidity also impact fiber formation. The distance between the needle tip and collector determines the extent of solvent evaporation from the nanofibers and deposition on the collector; collector rotation can result in nanofiber alignment during deposition [132]. Studies have shown that reducing the distance between the needle tip and collector increases the interconnectivity of the fibers [53]. In a study conducted by Beachley and Wen [156], a correlation was shown between increasing voltage and decreasing nanofiber diameters. They also showed that an increase in voltage leads to increased uniformity of the nanofibers. Increasing rotational speed of the drum collector also increases fiber alignment; Matthews and coworkers showed [157] that, at speeds of less than 500 rpm, randomly oriented collagen fibers were obtained. When the rotational speed was increased to 4500 rpm, the collagen fibers showed a significant increase in alignment (Figure 9).

Generally, nanofibers have high aspect ratios and large specific surface areas; depending on the chemical structure of the polymer, nanofibers may also have a high degree of flexibility and high mechanical strength [158]. Other mechanical properties such as Young’s modulus, yield stress, and tensile stress have also been shown to increase with higher rotational speed and fiber alignment [53,159]. Alignment of electrospun nanofibers is critical for some bioengineering/biomedical applications. Directed neurite growth has been achieved on aligned electrospun polycaprolactone (PCL) nanofibers, facilitating nerve regeneration in a rat sciatic nerve injury [160]. Additionally, aligned nanofibers provide guided electron transport pathways, have particular strain-induced electronic properties, have high electron and thermal diffusivity rates, and can be easily tailored to various morphologies [158]. Organic solar cells that employ aligned electrospun nanofibers could improve charge transport and increase power conversion efficiencies without any thermal post-treatments [129,161]. 

It is possible to increase electrospinning temperature by heating the polymer solution, using a controlled temperature chamber [162,163] or a heated syringe [164,165]. Increasing temperature decreases surface tension and solution viscosity, resulting in decreased fiber diameters [164,165]. However, this effect suffers from a competing effect: increasing temperature also accelerates solvent evaporation, which can terminate fiber stretching prematurely, increasing fiber diameters [164,166]. Thus, careful control of temperature is needed to control fiber diameters.

**Figure 10:**
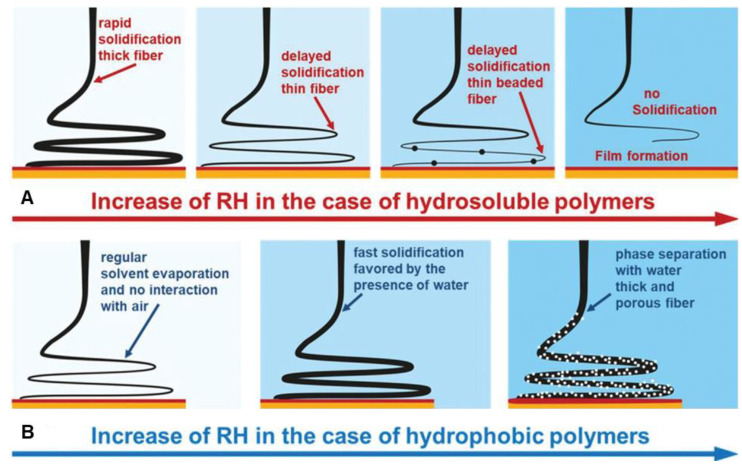
Effect of increasing relative humidity (RH) on (**A**) water-soluble and (**B**) hydrophobic electrospun polymers. Reprinted from [168]. Copyright 2021, John Wiley & Sons.

Humidity can also have profound effects on nanofibers [167,168]. How a polymer solution interacts with moist air depends on the solvent and on the polymer hydrophilicity [168,169]. At high relative humidity (RH), hydrophilic and hygroscopic polymers in nonaqueous solutions undergo slow solidification because of the high water content in the surrounding air, resulting in smaller fiber diameters; at very high RH, the jet thins until capillary instability results in bead formation (Figure 10A). Conversely, at high RH. water acts as a non-solvent for hydrophobic polymers, resulting in rapid solidification and larger fiber diameters; at very high RH, phase separation occurs, which results in formation of porous fibers (Figure 10B).

## 4. Electrospinning Conducting Polymers

### 4.1. Introduction

CP electrospinning has not received as much attention as electrospinning of other polymers that are easier to work with. CPs tend to have rigid backbones, low molecular weights, and low degrees of chain entanglement, which make them inadequate for electrospinning purposes [4,72,170,171,172]. There have been limited reports of successful electrospinning and nanofiber formation from pure CP solutions (“neat” electrospinning) [172,173,174,175,176]. Attempts to overcome these limitations by coaxial electrospinning, combining CPs with other, typically higher-molecular-weight polymers (“carrier polymers”) to electrospin as blends, or coating nonconductive polymer nanofibers to form layered structures are discussed in detail below. A comparison of advantages and disadvantages of the different methods can be found in Table 1.

### 4.2. Neat Electrospinning

Neat electrospinning of CPs is challenging to achieve. In addition to their rigid backbones and low molecular weights, many CPs are insoluble in organic solvents [72,173]. This makes the process of preparing and attaining neat CP solutions for electrospinning difficult; attempts to prepare high concentrations of CPs typically lead to precipitation, while low concentrations of CPs typically either do not form fibers at all, or they form beaded nanofibers. Nonetheless, researchers have been able to generate neat electrospun nanofibers from a variety of CPs including PPy [177], PANI [172,173,175], and P3HT [176]. Spectroscopic analysis of electrospun PANI blends indicated that PANI is stable to the high voltages used in electrospinning; no overoxidation of the PANI was observed [189]. In fact, individual fibers of electrospun PANI exhibited conductivity two orders of magnitude higher than solution-cast PANI films prepared from the same solution [175], likely due to significant chain alignment occurring during fiber formation. Similar results were observed for a low-molecular-weight polypyrrole doped with dodecylbenzenesulfonate [190]. Addition of a large excess of dodecylbenzenesulfonate increased chloroform solubility to enable electrospinning, and, after removal of excess dodecylbenzenesulfonate, the resultant PPy nanofiber conductivity was approximately double that of PPy powder or solution-cast films. 

**Figure 11:**
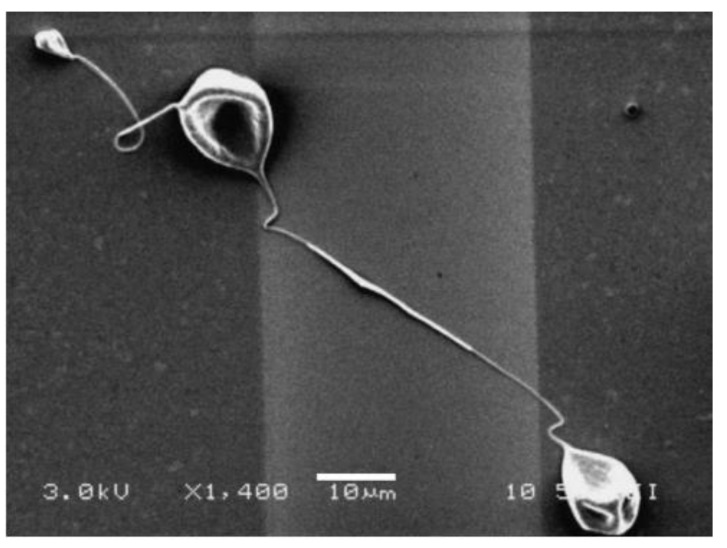
SEM showing a neat electrospun P3HT nanofiber having an average length of 54 µm and diameter of 670 nm [176]. Reprinted with permission from Ref. [176]. Copyright 2005 with permission from Elsevier.

González et al. [176] demonstrated early success electrospinning neat P3HT nanofibers. A 7 wt.% solution of regioregular P3HT was dissolved in chloroform and electrospun at 20 kV to yield nanofibers with average diameter of 670 nm (Figure 11); larger beads are also evident in the fibers. The high surface area of the nanofibers makes them particularly susceptible to reaction with atmospheric oxygen and water vapors; electrospinning and device fabrication in an inert environment might be necessary for implementation. Liu et al. [191] were able to reduce P3HT fiber diameters to ca. 180 nm by electrospinning 1 wt.% solutions of P3HT at 5.8 kV; the team noted the same issues with beading and with oxygen and water exposure.

A novel method using binary solvents for electrospinning neat poly [2-methoxy-5-(2-ethylhexyloxy)-1,4-phenylenevinylene] (MEH-PPV) was studied by Zhong et al. [192] They explored binary solvent mixtures of chloroform (a good solvent for MEH-PPV) and either methanol or isopropanol (poor solvents for MEH-PPV) and found that a lower surface tension and higher solution electrical conductivity favor fiber formation; thus, isopropanol was a better poor solvent than methanol to use in the binary mixtures. Using a 4:1 mixture of chloroform/isopropanol to dissolve MEH-PPV yielded bead-free nanofibers, while increasing chloroform content even slightly (to 5:1 or higher) yielded beaded nanofibers. Researchers hypothesized that addition of isopropanol caused polymer chain aggregation, increasing interchain interactions to the point that steric hindrance of the MEH-PPV backbone motions increased effective conjugation lengths [192,193,194], stabilizing physical (chain) entanglements and enabling electrospinning of the neat MEH-PPV solution without addition of a carrier polymer [192,195]. For aligned MEH-PPV nanofibers, the researchers were also able to use polarization anisotropy spectroscopy to determine that the MEH-PPV chains were preferentially aligned along the electrospun fiber axis.

### 4.3. Coaxial Electrospinning

Coaxial electrospinning has emerged as a successful technique to obtain CP-containing nanofibers. Coaxial electrospinning produces core–sheath-structured nanofibers by forming a fiber that consists of complementary materials in the core and sheath [196]. Coaxial electrospinning enables traditionally non-electrospinnable CPs to be able to be electrospun with an adjacent electrospinnable polymer. Two styles of coaxial electrospun nanofibers can be generated: (i) the core comprises a CP with the spinnable polymer as the post-process removable shell, or (ii) the spinnable polymer is the core, while the CP is the shell [173]. Additional parameters must be considered for the coaxial electrospinning process, including compatibility of the solutions and needle diameter ratios [184]. Coaxial electrospinning has provided a successful alternative for researchers to obtain neat CP electrospun nanofibers, after simple post-treatment processes to remove the nonconductive polymer. An in-depth review of coaxial electrospinning and its applications was recently published by Steckl and Han [196] and is recommended reading for further insights. A summary of coaxial electrospinning references discussed below can be found in Table 2.

**Figure 12:**
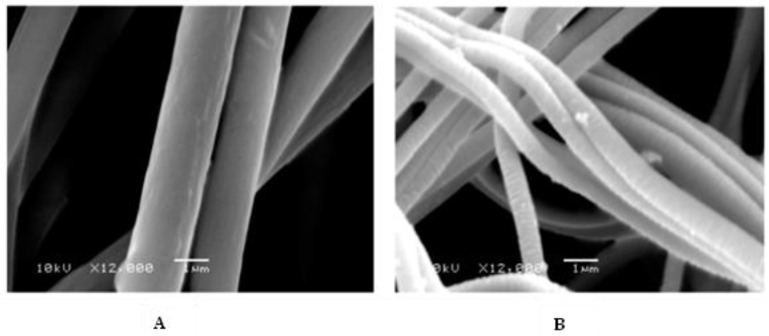
SEMs of (**A**) core–sheath PANI/PMMA electrospun nanofibers and (**B**) electrospun PANI nanofibers after removal of PMMA [182]. Reprinted with permission from Ref. [182]. Copyright 2012.

When blends of PANI and PMMA would not electrospin due to insufficient elasticity of the polymer solutions, Zhang and Rutledge [182] utilized coaxial electrospinning to generate PANI/PMMA nanofibers, using emeraldine base PANI (M_w_ = 65,000 Da) dissolved in chloroform for the inner core and poly(methyl methacrylate) (PMMA, M_w_ = 540,000 Da) dissolved in N,N-dimethylformamide (DMF) as the outer shell. Smooth and continuous PANI/PMMA nanofibers were obtained with diameters ca. 1440 nm. The PMMA outer shell could be removed via immersion in an isopropanol solution for 1 h to yield neat PANI nanofibers with decreased diameters of ca. 620 nm (Figure 12) and conductivities of 50 S·cm^−1^. 

Chen and coworkers used coaxial electrospinning to prepare nanofibers with P3HT cores and PMMA shells [197]. The PMMA shell could be removed after electrospinning by immersion in acetone. The researchers evaluated three different chlorinated solvents to prepare the P3HT solutions for electrospinning and found that chloroform and chlorobenzene, both good solvents for P3HT, allowed for chain stretching during electrospinning. This increased alignment of adjacent chains, producing highly oriented crystalline grains and smaller crystal sizes as compared to P3HT electrospun from 1,2,4-trichlorobenzene (TCB). This higher orientation resulted in a three-orders-of-magnitude increase in charge carrier mobility (from 10^−4^ to 10^−1^ cm^2^·V^−1^·s^−1^) for the fibers prepared from chloroform and chlorobenzene relative to those prepared from TCB. P3HT nanofibers prepared from chloroform offered the additional benefit of enhanced ductility relative to the fibers prepared from chlorobenzene or TCB.

Coaxial needles can be used in the preparation of electrospun CP nanofibers in ways other than the typical ones described above. In a slightly different approach to electrospinning using coaxial needles, Lee and coworkers [198] conducted a study on electrospinning of P3HT (M_w_ = 87,000 Da) in CHCl_3_ at a concentration of 11–13%; at lower concentrations, beaded fibers formed. The researchers observed that, during electrospinning of neat P3HT, the needle tip became blocked after about 10 s, due to the P3HT crystallizing as the solvent evaporated. To combat this, they utilized a coaxial electrospinning setup in which additional CHCl_3_ was delivered via the outer, nonconductive nozzle, while P3HT solution in CHCl_3_ was delivered via the inner, conductive nozzle. Smooth, uniform P3HT nanofibers with an average thickness of ~500 nm were obtained. The coaxial setup allowed for neat P3HT nanofibers to be generated without the aid of a carrier polymer and with no adverse impacts to the electrical properties of the CP used.

**Table 2:** Coaxially electrospun CPs discussed in the text.

Core	Shell	Core Solvent	Shell Solvent	Electrospinning Parameters				Fiber Diameter	Other Properties	Conductivity	Application	Ref	Notes
				Applied Voltage	Core Flow Rate	Shell Flow Rate	Tip-to-Collector Distance						
P3HT	CHCl_3_	CHCl_3_	CHCl_3_	18 kV	0.7 L/min	0.5 L/min	7 cm	~500 nm	Field effect mobility: 0.017 cm^2^/V-s	-	FET device fabrication	[198]	Field effect mobility of P3HT/PCL blend fibers: 0.00047 to 0.0012 cm^2^/V-s
PANI	PMMA	CHCl_3_/DMF	DMF	34 kV	0.01 mL/min	0.05 mL/min	30 cm	620 ± 160 nm	-	50 ± 30 to 130 ± 40 S/cm (impedance)		[182]	Conductivity of PANI/PEO electrospun blend: 0.02 ± 0.01 to 8.1 ± 3.0 S/cm, PANI/PMMA electrospun blend: 2.0 ± 1.0 × 10^−5^ to 2.3 ± 1.6 × 10^−2^ S/cm, PMMA removed after electrospinning
MEH-PPV/PCBM	Polyvinylpyrrolidone (PVP)	Chlorobenzene	8.5:1.5 EtOH:H_2_O	-	4 µL/min	25 µL/min	11 cm	~800 nm	-	-	Solar cells	[199]	Mix of blend and coaxial electrospinning, PVP removed after electrospinning
P3AT	PMMA	CHCl_3_	Chloro-benzene	8.1–10 kV	0.1 mL/h	1.0 mL/h	13 cm	144–414 nm	Field effect mobility: 1.54 × 10^−4^ to 1.62 × 10^−1^ cm^2/^V-s	-	FETs	[200]	PMMA removed after electrospinning
PU	PANI/PVA	CHCl_3_	H_2_O/SDS/Acetic acid	-	1×	1–10× core	13 cm	60 nm (core), 98–100 nm (shell)	Elongation at break: 248.3 ± 19.3 to 395.3 ± 51.1%	0.019 ± 0.002 to 1.092 ± 0.272 × 10^−3^ S/cm (Impedance)	Wearable pH sensor	[201]	
AgNO_3_	poly [2,7-(9,9-dihexylfluorene)-alt-4,7-(2,1,3-benzothiadiazole)] (PFBT)/PMAA	Ethyl glycol	THF (PFBT), 1:1 H_2_O:DMF (PMAA)	14–15 kV	0.1 mL/h	1.0 mL/h	13 cm	550 nm (PFBT NPs: 13.33 ± 4.18 nm)	-	-	Organic photovoltaic cells	[202]	

### 4.4. Co-electrospinning with Carrier Polymers 

Typically, neat CP solutions for electrospinning must be at a low concentration to facilitate the dissolution of the CP chains. These low-concentration CP solutions are often not viscous enough for electrospinning due to lack of chain entanglement. A carrier or host polymer is a high-molecular-weight polymer that is used in electrospinning to help an electrospinning solution reach an adequate viscosity for electrospun nanofiber formation. High-molecular-weight carrier polymers reach chain entanglement at low concentrations; hence, the addition of small amounts of high-molecular-weight carrier polymer can enable electrospinning of CPs with minimal impact on CP properties [198]. Thus, CPs are often electrospun using carrier polymers, including polyacrylonitrile (PAN), poly(ethylene oxide) (PEO), poly(vinyl chloride) (PVC), poly(methyl methacrylate) (PMMA), (PS), and poly(lactic-*co*-glycolic acid) caprolactone) (PLGA), as shown in Figure 8. A summary of co-electrospinning approaches discussed in this review can be found in Table 3.

One disadvantage of electrospinning using these carrier polymers is that, while the carrier polymers impart beneficial properties, at higher carrier polymer concentrations, the resultant nanofiber blends may not possess the desired CP properties, such as conductivity or electrochromism. In addition to increasing insulating content, electrospinning CPs with carrier polymers may also introduce surfactants or byproduct impurities [192].

The research group of Nobel Prize winner Alan MacDiarmid conducted some of the early investigations into electrospun CPs utilizing carrier polymers or coating nonconductive nanofibers. They electrospun blends of doped PANI with PEO (50–89%) [189] or PS (80%) [175] as a carrier polymer. Fiber diameters varied with the concentration and nature of the carrier polymer; while PANI/PEO blends yielded fiber diameters from 0.9 to 1.9 µm [189], PANI/PS blends yielded fiber diameters from 72 to 100 nm [175]. Conductivity was found to increase as concentration of PANI in the blends increased, and conductivities of fibers were slightly lower than conductivities of cast films of the same blends [189].

More recently, co-electrospinning of PEDOT:PSS was accomplished with a variety of carrier polymers [92]. Addition of very-high-molecular-weight (4,000,000 Da) PEO at levels as low as 0.5% was sufficient to cause PEDOT:PSS to electrospin into non-beaded fibers [203]. Fiber diameters varied from 300 to 400 nm depending on electospinning conditions, and electrical conductivity of single fibers reached 35 S·cm^−1^.

**Table 3:** Summary of reports of electrospinning using carrier polymers.

ICP	Carrier Polymer	Solvent	Electrospinning Parameters			[ICP]	Fiber Diameter	Other Properties	Conductivity	Application	Ref	Notes
			Applied Voltage	Flow Rate	Tip-to-Collector Distance							
PPy	PCL/gelatin (1:1)	Hexafluoroisopropanol (HFIP)	12 kV	1 mL/h	10 cm	15%	216 ± 36 nm	Contact angle: 46.9 ± 2.0°, Young’s modulus: 16.8 ± 1.9 MPa, elongation at break: 13.6 ± 3.2%	0.013 mS/cm (4-point probe)	Cardiac tissue regeneration	[50]	-
						30%	191 ± 45 nm	Contact angle: 63.5 ± 2.8°, Young’s modulus: 50.3 ± 3.3 MPa, elongation at break: 3.7 ± 1.4%	0.37 mS/cm (4-point probe)			-
PEDOT:PSS	PVP	EtOH	1.6 kV	-	4.0 cm	37%	800 nm	-	10^5^ Ω-m (two-metal-microprobe impedance)	Gas sensor	[81]	Centrifugal electrospinning
			20 kV	-	10 cm		600–800 nm	-	-			Electrospinning
PEDOT:PSS	PEO	DMF	8.5 kV	0.2 mL/h	10 cm	1.30%	105–157 nm	-	13–39 kΩ (sheet resistance)	-	[95]	-
PEDOT	PCL	CHCl_3_: acetone (2:1)	15 kV	10 mL/h	15 cm	0.60%	3900 ± 700 nm	Contact angle: ~115°		Drug delivery system	[142]	With curcumin
						0.60%	5400 ± 1200 nm	Contact angle: ~125°				Without curcumin
Polyindole (Pind)	PEO	CHCl_3_	20 kV	1 mL/h	15 cm	80%	-	Specific capacitance: 322–555 F/g	-	Energy storage	[144]	Blend electrospinning–electrospraying with added carbon nanotubes
PPy	PEO	H_2_O	30 kV	-	20 cm	37.5–71.5%	200–300 nm, 120–220 nm with non-ionic surfactant	-	4.9 × 10^−8^ to 1.2 × 10^−5^ S/cm (two-point method)	-	[177]	-
	none	DMF	30 kV	-	20 cm	100%	70 nm	-	2.7 × 10^−2^ S/cm (two-point method)	-		Doped with di(2-ethylhexyl) sulfosuccinate sodium salt (DEHS)
	PEO	H_2_O	30 kV	-	20 cm	37.5–50%	100–150 nm	-	1.1 × 10^−4^ to 3.5 × 10^−4^ S/cm (two-point method)	-		PPy-SO3H doped with DEHS
PANI	PVA	H_2_O	10–20 kV	0.2–0.8 mL/h	10 cm	10%	234–560 nm	-	-	-	[174]	Studies done on effect of electrospinning parameters on nanofiber diameter
MEH-PPV	PCL	CHCl_3_/DMF	10–22 kV	0.015–0.02 mL/min	15 cm	1%	300–1100 nm	-	-	-	[192]	-
P3HT	PCL	CHCl_3_	18 kV	-	7 cm	10–90%	~30–250 nm	-	-	FET device fabrication	[198]	PCL dissolved with CHCl_3_ in electrospun mat
PANI	PEO	CHCl_3_/DMF	32–40 kV	0.015–0.05 mL/min	30 cm	11–67% PANI	1200 ± 300 to 2700 ± 900 nm	-	0.02 ± 0.01 to 8.1 ± 3 S/cm (impedance)	-	[182]	-
	PMMA	DMF	25–31 kV	0.04–0.05 mL/min	30 cm	3.8–25% PANI	1500 ± 200 to 1900 ± 400 nm	-	2.0 ± 1.0 × 10^−5^ to 2.3 ± 1.6 × 10^−2^ S/cm (impedance)	-		Coaxially electrospun PANI/PMMA has conductivity of 50 ± 30 S/Cm
PANI	PEO	CHCl_3_	25 kV	-	25 cm	11–50%	950–1900 nm (average: 1600 nm)	-	~0.00001–0.1 S/cm (4 point probe)	-	[189]	Conductivity of film of same composition: ~0.001–1 S/cm
PANI-HCSA	PS	CHCl_3_	-	-	-	20% PANI	72–100 nm (average: 85.8 nm)	-	Enough to be observed bare on an SEM	-	[175]	Pure electrospun PANI in H_2_SO_4_ properties: Fiber diameter: 96–275 nm, conductivity: ~0.1 S/cm
PEDOT:PSS	PEO	-	3–5 kV	28–625 mg/h	-	27–65%	297–441 nm	-	0.001–35.5 S/cm (2-electrode conductivity cell)	-	[203]	-
PDBTT	PCL	75:25 CHCl_3_:MeOH	15 kV	1 mL/h	12 cm	6.25% PDBTT	350 ± 69 nm	Contact angle: 72 ± 2°, Young’s modulus: 20 ± 1 MPa, elongation at break: ~200%	-	Skin tissue engineering	[204]	-
PPy	PEO, PCL	DMSO, CHCl_3_, 2-chloroethanol	20 kV	0.5 mL/h	15 cm	40% PEO-PPy	120 ± 30 nm	Young’s modulus: 108 ± 3.2 MPa to 115 ± 4.1 MPa, elongation at break: 40.1 ± 2.7 to 46.6 ± 3.4%	0.0009–0.002 S/cm (4 point probe)	Tissue engineering	[205]	PEO modified with PPy. Conductivity of PEO-PPy: 0.24–0.31 S/cm, conductivity of PPy: 0.59 S/cm
PANI	PAN	DMF	18 kV	0.5 mL/h	15 cm	1–3%	200–600 nm	-	-	Photovoltaics	[206]	-
PPy							250–800 nm	-	-			-
PT							200–650 nm	-	-			-
PPy	PVDF	DMF/Acetone	10 kV	0.7 mL/h	14 cm	3%	325 ± 143 nm	Contact angle: 108.6 ± 0.1°, Young’s modulus: 27.4 MPa, capacitance: 3.59 × 10^−10^ F	-	Biomaterials	[207]	-
PANI							390 ± 138 nm	Contact angle: 111.0 ± 0.1°, Young’s modulus: 29.9 MPa, capacitance: 1.27 × 10^−10^ F	-			-
PANI-L-glutamic acid							2512 ± 1182 nm	Contact angle: 112.5 ± 0.3°, Young’s modulus: 35.6 MPa, capacitance: 4.03 × 10^−10^ F	-			-
PANI-Emeraldine Base	PLGA	HFIP	12.3–13.6 kV	1 mL/h	-	4–8%	58.9 ± 14.2 to 184.7 ± 31.9 nm	Young’s Modulus: 91.7 ± 5.1 MPa (6% PANI)	9.5 × 10^−7^ to ~10^−3^ S/cm (4-point probe)	Cardiac tissue engineering	[208]	-
PANI	PCL	HFIP	14–18 kV (negative voltage: 0.2–2.5 kV)	1 mL/h	10 cm	0.3–3%	-	-	1.34 ± 0.015 to 8.76 ± 0.02 uS/cm (solution)	Biomaterials	[209]	-
MEH-PPV, P3HT	PVP	CHCl_3_	8 kV	0.5 mL/h	15 cm	37.50%	300–450 nm	-	1.02–1.34 × 10^−7^ S/m (tunneling AFM)	Solar cells	[210]	Blend of MEH-PPV and PCBM electrospun with PVP. P3HT/PCBM added with spin coating
PEDOT:PSS	PEO	H_2_O/DMF/Triton X-100	18 kV	1.5 uL/min	15 cm	8%	2000 nm	-	1.8 S/cm (4-point probe)	Flexible, transparent supercapacitors	[211]	Mixed, then excess PEO removed with ethylene glycol. Conductivity of film of similar composition: 290–650 S/cm (4-point probe)
PANI	PEO	CHCl_3_	5 kV	0.6 mL/h	25 cm	93%	678 ± 54 nm	-	0.114 S/cm (EIS)	Supercapacitor	[212]	-
						81%	491 ± 86 nm	-	0.154 S/cm (EIS)			12% CN%
PANI	PEO	CHCl_3_	7 kV	0.5 mL/h	7 cm	70%	500–2000 nm	Specific capacitance: 235.2 F/g	-	Hybrid capacitor	[213]	Mixed, then CNTs and activated carbon also electrosprayed. 1000–5000 nm nanoparticles
Pind	PEO	CHCl_3_	15 kV	0.5 mL/h	15 cm	90%	100–1400 nm (average 669)	Specific capacitance: 155–238 F/g	-	Supercapacitor	[214]	-
						80%	Average 726 nm	Specific capacitance: 476–521 F/g	-			10% CNT
Regioregular P3HT	PLA	CHCl_3_	9 kV	2 mL/h	-	37–44%	100–4000 nm	-	-	Sensors	[215]	-
PANI	PCL	HFIP	5.8 kV	0.3 mL/h	55 cm	9–20%	145 ± 14 to 162 ± 27 nm	-	4.1 × 10^−5^ S/cm to 7.0 × 10^−4^ S/cm (I–V plots)	H_2_O vapor, NO_2_ sensor	[216]	PANI dispersed in HCSA, then mixed with PCL
PANI	PVP	1:1 EtOH:DMF	17 kV	0.4 mL/h	20 cm	-	>200 nm	-	-	H_2_ sensor	[217]	PANI/CSA mixed with SnCl_2–_2H_2_O, Al(NO_3_)_3–_9H_2_O, PVP, then PVP calcined
Novel Need to be able poly(fluorenylene ethynylene)s-co-polythiophenes	PS	3:1 DMF:THF	20 kV	1 mL/h	25 cm	10%	200–800 nm	-	-	Nitroaromatics sensor	[218]	-
PEDOT:PSS	PVP	DMF	18 kV	0.2 mL/h	15 cm	80%	165.4 ± 58.0 nm to 171.8 ± 36.7 nm	-	-	Volatile organics sensor	[219]	PEDOT:PSS mixed with PVP and Multi-walled carbon nanotubes-COOH
PANI/ poly(thiophene) based polymer	PMMA	N-methyl-2-pyrrolidone	20 kV	0.6 mL/h	10 cm	2%	500 nm	-	-	N-butanol sensing	[220]	-
PFO	PMMA	CHCl_3_	20 kV	0.3 mL/h	6 cm	0.1–2%	619 ± 14 nm	-	-	CHCl_3_ testing	[221]	-
PANI	P3HB	4:1 CHCl_3_:DMF	15–28 kV	3–17 µL/min	20 cm	-	452.9 ± 31.8 to 696.7 ± 185.3 nm	-	-	Ethanol sensor	[222]	PANI on SnO_2_ nanorods, with either Ni or Pd
PPV	PVA	H_2_O	10 kV	0.3 mL/h	10 cm	1%	Around 200 nm	Contact angle: 85–131°	-	Aromatic organic solvent detection	[223]	Prepolymer blended with PVA and then electrospun, and then thermal treatment to convert to polymer
PANI	PS	CHCl_3_	30 kV	-	30 cm	15%	800–1000 nm	-	-	Glucose sensor	[224]	-
PANI	PEO	1:1 EtOH:CHCl_3_	10 kV	0.2 mL/h	10 cm	8%	250–500 nm	-	10^5^ to 10^7^ Ω (impedance)	Humidity sensor	[225]	Conductance of film of same composition: 10^6^ to 10^7^ Ω (Impedance)
PT	PAN	DMF	12 kV	0.01 mL/h	8 cm	3–23%	600 ± 200 to 1300 ± 700 nm	-	-	Phosphate sensor	[226]	-
PANI	Polyvinylbutyral, polyamide-6	Formic acid	27 kV	0.6 mL/h	15 cm	20%	326 nm	-		Hg sensing	[227]	-
PPV	PVA	H_2_O	12 kV	0.3 mL/h	10 cm	-	250 nm	-	-	Sudan dye detection	[228]	Prepolymer and PVA electrospun, then converted to polymer with heating
PPy	PEO	EtOH/H_2_O	16 kV	-	14 cm	50–80%	100–600 nm	-	2.28 × 10^−7^ to 2.54 × 10^−5^ S/cm (I–V curves)	IgG sensing	[229]	-
PEDOT:PSS	PVA	H_2_O	20 kV	1.4 mL/h	10 cm	18%	150–200 nm	Contact angle: 68°, Young’s modulus: 232 MPa, elongation at break: 11%	0.88 mA (electrochemical current)	Carcinogen detector	[230]	-
PANI	PU, PVA	Core: CHCl_3_, Shell: H_2_O/SDS/Acetic acid	-	1:1–10:1 shell:core flow rate	13 cm	56%	60 nm (core), 98–100 nm (shell)	Elongation at break: 248.3 ± 19.3% to 395.3 ± 51.1%	0.019 ± 0.002 to 1.092 ± 0.272 × 10^−3^ S/cm (impedance)	Wearable pH sensor	[201]	Coaxial electrospinning of a PU core and a blended PANI/PVA shell
							-	Elongation at break: 300.5 ± 103.4%	4.577 ± 0.472 S/cm (impedance)			Blend electrospun fibers
PEDOT:PSS	PEO	H_2_O	15 kV	0.1 mL/h	15 cm	10%	-	-	Up to 200 S/cm change from unprocessed nanofibers (from I–V curves)	Wearable flex sensor	[231]	-
PEDOT:PSS	PEO	H_2_O	23 kV	0.1 mL/h	15 cm	5%	-	-	13.82 mS/cm	-	[232]	-
PANI	PAN	DMF	20 kV	1 mL/h	10 cm	10–40%	153–190 nm	Contact angle: ~150°, Young’s modulus: 12.64 ± 2.91 MPa, elongation at break:	-	Oil/H_2_O emulsion separation	[233]	Core/shell electrospun nanofibers of same composition had the following properties: fiber diameter: 333 nm, contact angle: 150°, Young’s modulus: 302.8 ± 78.30 MPa, elongation at break: 47% ± 1%
PEDOT:PSS	PEO	H_2_O	22 kV	1 mL/h	11 cm	4.5–6.5%	106 ± 49 to 157 ± 65 nm	-	3.09 × 10^–5^ to 6.10 S/cm (4-point probe)	Toxic protein removal	[234]	Blend of multiwalled carbon nanotubes, PEDOT:PSS, PEO, (3-glycidyloxypropyl)trimethoxysilane electrospun
PCZ	PVDF	DMF	20 kV	1 mL/h	15 cm	4%	650 ± 80 nm	Contact angle: 96°, Young’s modulus: 20.3 MPa, elongation at break: 24%	2.33 × 10^–4^ S/cm (2-point probe)	Human motion energy harvesting	[235]	-
PANI							820 ± 180 nm	Contact angle: 94°, Young’s modulus: 22.8 MPa, elongation at break: 14%	1.61 × 10^–4^ S/cm (2-point probe)			
PEDOT:PSS	PVA	DMSO	23 kV	0.4 mL/h	9 cm	5%	440 nm	Young’s modulus: 1.34–7.47 MPa, elongation at break: 2.99–9.93%	0.67–41.5 S/cm (4-point probe)	Flexible thermoelectric generator	[236]	Blend electrospun then dip-coated with PEDOT:PSS, then coated with AgNPs
PEDOT:PSS	Poly(N-isopropyl acrylamide-co-N-methylolacrylamide)	H_2_O	20 kV	0.1 mL/h	12.5 cm	3.8–19.9%	-	-	0.085–11.2 S/cm (voltmeter)	Thermoresponsive composite	[237]	-
						Thin film	-	-	0.084–192.3 S/cm (voltmeter)			
PANI	PVA	H_2_O	20 kV	1.5 mL/h	16 cm	0.2–0.6%	400–730 nm	Elongation at break: ~35–60%		Electrochromic device	[238]	Polymerization of PANI in solution with PVA, then electrospinning of blend
PANI	Silk fibroin	Formic acid	15–30 kV	0.1–0.8 mL/h	12 cm	2.5–30%	~75–230 nm	Contact angle: ~107–120°, Elongation at break: ~1.5–3.75%	up to 0.5 S/cm (4-point probe)	Conductive textiles	[239]	-
PPy	PMMA	DMF	15 kV	15 µL/h	5 cm	7.30%	1170 ± 400 to 1980 ± 500 nm	-		Electrochromic device	[240]	PPy electrochemically polymerized in ionic liquid, then dissolved and mixed with PMMA
poly[(9,9-bis(3′-(N,N-dimethylamino)propyl)-2,7-fluorene)-alt-2,7-(9,9-dioctyl-fluorene)]	Acrylonitrile butadiene rubber	1:2 DCM: chlorobenzene	13.2 kV	0.1 mL/h	19 cm	3–7%	422 ± 117 to 1370 ± 300 nm	-		Wearable electronics	[241]	-

### 4.5. Coating with Conducting Polymers

Coating of electrospun nanofibers of commodity polymers (Figure 8) with CPs has become a highly active area of research due to potential applications in smart textiles, energy storage, and sensors. Various techniques for coatings are employed, such as dip coating, vapor phase deposition, and in situ polymerization [242,243]. By coating the electrospun nanofibers with CPs such as PPy, PEDOT, PANI, or PEDOT:PSS, a conductive textile is created that combines the mechanical properties of the electrospun polymer with the electronic properties of the CP. It is important to note that some researchers have found that, while the mechanical properties of the layered nanofibers are improved relative to pristine CPs, filling the fiber interstices with stiff CP may reduce breaking strain while enhancing ultimate stress and modulus [90,244]. A summary of reports of CP coatings on electrospun polymers can be found in Table 4.

**Figure 13:**
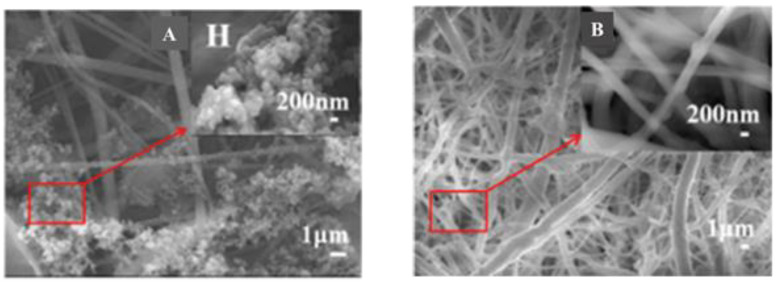
SEMs of (**A**) cellulose/PNVPy coated nanofibers and (**B**) cellulose/P3HT-coated nanofibers [245]. Republished with permission from Ref. [245], Permission conveyed through Copyright Clearance Center Inc.

Chemical compatibility must be considered when coating polymer nanofibers with CPs. Zha et al. [245] electrospun cellulose nanofibers and then used an in situ polymerization method to generate a coating of poly(N-vinylpyrrole) (PNVPy) for one set of fibers and a coating of P3HT on another. Both types of electrospun CP-coated nanofiber mats were found to exhibit favorable cell adhesion, proliferation and cytocompatibility with PC12 cells. However, PNVPy formed aggregated nanoparticles on the electrospun cellulose nanofibers while P3HT formed a continuous coating (Figure 13). This difference was apparent in the performance of these two systems: cellulose/P3HT demonstrated increased hydrophilicity and more effective PC12 cell proliferation relative to cellulose/PNVPy. Regardless, both types of electrospun CP coated nanofiber mats showed positive outcome toward neural tissue engineering.

PEDOT was oxidatively polymerized onto electrospun nanofiber blend of nitrile butadiene rubber (NBR) and poly(ethylene glycol) dimethacrylate (PEGDMA) for bioelectronic sensor applications as demonstrated by Fallahi et al. [244] An increase in the overall elastic modulus of the PEDOT coated NBR/PEGDMA nanofiber mats was observed at ca. 3.8 MPa compared to uncoated NBR/PEGDMA nanofiber mats at just 0.7 MPa. Inversely, PEDOT-coated NBR/PEGDMA nanofibers mats exhibited a threefold decrease in elongation strain, 75% maximum strain compared to 122% maximum strain for uncoated NBR/PEGDMA nanofiber mats. For all electrospun nanofiber mats there was no significant reduction in hysteresis after 10 cycles at 20% elongation; more specifically, the PEDOT coated NBR/PEGDMA nanofibers mats showed stabilizing behavior even after 100 cycles. Mechanical and electrical stability measurements were conducted on aligned PEDOT-coated NBR/PEGDMA nanofibers mats; the change in resistance was measured over 100 extension and relaxation cycles at 20% strain rate. The conductivity was measured at ca. 6 S·cm^−1^; nanofiber alignment led to anisotropic electrical connectivity, creating stable and reversible changes in resistance. In addition to imparting electrical properties, the PEDOT coating also acts a reinforcing agent that leads to an increase in elastic modulus and improved shape recovery forces after stress removal. There was a decrease in flexibility due to the addition of PEDOT, but flexibility and stretching properties were still within favorable ranges for the purposes of this application.

In another example, electrospun polyurethane (e-PU) nanofibers were dip-coated with PEDOT:PSS as shown by Ding et al. [246] The effects of using one to three dip coatings were explored. The team investigated the conductivity effects when the conductively coated fabrics were stretched, twisted, and bended, and all demonstrated sufficiently stable conductivity to light up a small light-emitting diode (LED). The conductivity of the e-PU nonwovens increased relative to the number of dip coatings applied; one PEDOT:PSS dip coating yielded a conductivity of 0.3 S·cm^−1^, while a second dip coating increased conductivity to 0.9 S·mm^−1^, and a third dip coating increased conductivity to 2 S·cm^−1^. Conversely, tensile strength saw a decrease in elongation to break as more dip coatings were applied, uncoated e-PU and one PEDOT:PSS dip coating exhibited ca. 400% elongation at break, while two dip coatings decreased elongation at break to ca. 230%, and three dip coatings reduced elongation at break to ca. 40%. This dip coating method produced PEDOT:PSS/PU fabrics exhibiting highly stable conductivities as stretchable conductors for flexible electronics comparable to those in recent literature [246,247].

**Figure 14:**
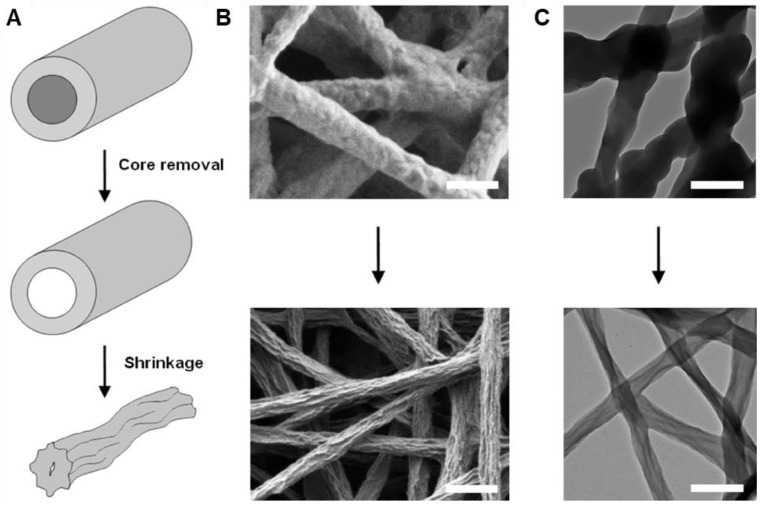
Polyvinylpyrrolidone electrospun with ferric tosylate and coated with PEDOT is rinsed with methanol to form hollow PEDOT nanofibers that collapse somewhat and fuse together [186]. (**A**) Process upon rinsing with methanol. (**B**) SEM and (**C**) TEM of the nanofibers. All scale bars represent 1 μm. Reprinted with permission from Ref. [186].

In a slightly different approach, solutions of PMMA [248] or poly(acrylic acid) (PAA) [249] with the EDOT monomer were electrospun into aqueous oxidant solutions. This resulted in formation of EDOT-containing PMMA or PAA fibers that underwent subsequent EDOT polymerization upon heating to yield fiber mats with conductivities up to 1 S·cm^−1^. It is also possible to co-electrospin polymers with oxidant and then expose the resultant fibers to monomer to produce CP coatings. For instance, poly(N-vinylpyrrolidone) (PVP) was electrospun with ferric tosylate to form fiber with average diameter of 600 nm [186]. PEDOT was then deposited on the resultant nanofibers via vapor-phase polymerization, increasing the average fiber diameter to 710 nm. Subsequent rinsing with methanol removed PVP and residual oxidant, collapsing the PEDOT fibers slightly and decreasing average fiber diameter to 350 nm (Figure 14). The PEDOT fibers appeared to fuse together during the rinsing process to yield fiber mats with excellent conductivity, ca. 60 S·cm^−1^.

**Table 4:** Summary of efforts to coat electrospun polymers with CPs.

ICP	Electrospun Polymer	Method	Solvent	Electrospinning Parameters			Nanofiber Diameter	Conductivity (Technique)	Other Properties	End Application	Ref.	Notes
				Applied Voltage	Flow Rate	Tip-to-Collector Distance						
PPy	75/25 PLGA	In situ chemical oxidative polymerization	HFIP	15 kV	3 mL/h	15 cm	430 ± 180 to 520 ± 150 nm (total), 85 ± 41 nm (shell)	7.4 ± 3.2 × 10^3^ to 9.0 ± 6.0 × 10^4^ Ω-s/square (surface resistivity)	-	Neural tissue engineering	[51]	-
PANI	PAN	In situ chemical oxidative polymerization	DMF	10 kV	0.8 mL/h	-	667.0 nm	-	Young’s modulus: ~475 MPa (initial modulus, PANI), elongation at break: ~10%	Gas sensor	[90]	Novel electrospinning device
PPy	PU	In situ chemical oxidative polymerization	TFA	-	-	-	80–808 nm, 60–90 nm (shell)	~0.47 μS/cm (4-point probe	Reduced hydrophilicity with PPy coating	Nerve tissue engineering	[145]	-
	Chitosan-PU							~0.45 μS/cm (4-point probe)				
		In situ polymerized on electrospun CS-PU/functionalized MWCNT						~2.7 μS/cm (4-point probe)				
PEDOT:Tosylate	PVP	Electrospinning PVP with oxidant, vapor phase polymerization with EDOT, then dissolving PVP in EtOH	Butanol/IPA	20 kV	2 mL/h	15 cm	700 nm (PEDOT:Tos)	Not measured but stable and dependent on strain	Elongation at break hypothesized to be 140%	-	[171]	Hollow shell
Poly(N-(methacryl ethyl) pyrrole)	Hydrolyzed cellulose	Alkaline hydrolysis of electrospun substrate then in situ chemical oxidative polymerization	-	18 kV	0.015 mL/min	20 cm	531 ± 230 nm		Contact angle: 43.34 ± 0.77°, Young’s modulus: 0.564 ± 0.063 MPa, elongation at break: 6.401 ± 0.142%	Nerve regeneration	[242]	-
Poly(N-(2-hydroxyethyl) pyrrole)							804 ± 265 nm		Contact angle: 32.14 ± 0.69°, Young’s modulus: 0.205 ± 0.074 MPa, elongation at break: 11.984 ± 0.879%			
Poly(3-(ethoxycarbonyl) thiophene)							680 ± 231 nm		Contact angle: 89.21 ± 0.88°, Young’s modulus: 0.537 ± 0.096 MPa, elongation at break: 13.610 ± 0.527%			
Poly(3-thiophenethanol)							653 ± 245 nm		Contact angle: 35.24 ± 1.84°, Young’s modulus: 0.417 ± 0.026 MPa, elongation at break: 4.188 ± 0.217%			
Poly(N-vinylpyrrole)	Hydrolyzed cellulose	Alkaline hydrolysis then in situ chemical oxidative polymerization	THF/DMF	18 kV	-	20 cm	703 nm		Contact angle: 18.06–24.59°	Nerve regeneration	[245]	-
Poly(3-hexylthiophene)							1130 nm		Contact angle: 41.98–58°			
PEDOT	Nitrile butadiene rubber, Poly(ethylene glycol dimethacrylate)	Crosslinking while electrospinning, then allowed to crosslink more, then in situ chemical oxidative polymerization of EDOT	CHCl_3_	-	1 mL/h	10 cm	~15,000 ± 500 nm	4.6–5.8 S/cm (4-point probe)	Young’s modulus: 3.8–10.3 MPa, elongation at break: 48.3–75.1%	Muscle contraction and movement sensor	[244]	-
PEDOT:PSS	PU	Dip coating in PEDOT:PSS	DMF	15 kV	0.8 mL/h	30 cm	-	29.7–200 S/m (sheet resistance)	Elongation at break: 40–230%	Stretchable conductors	[246]	-
PEDOT	PMMA	PMMA electrospun with EDOT, then electrospun onto aqueous oxidant	DMF	6–7 kV	0.9 mL/h	-	50 nm layer of PEDOT	0.19–7.6 S/cm (4-point probe)	-	-	[248]	-
PEDOT	PAA	PAA electrospun with EDOT then oxidative polymerization	DMF	14 kV	7 mL/h	18 cm	298.6–343.5 nm (~53.1–160.3 nm PEDOT coating)	0.006–0.16 S/cm (4-point probe)	-	-	[249]	-
PEDOT	PVP	PVP electrospun with oxidant and then vapor phase polymerization in chamber with EDOT, then dissolving PVP in MeOH	Butanol	27 ± 1 kV	-	15 cm	350 ± 60 nm	60 ± 10 S/cm (4-point probe)	-	-	[250]	Hollow shell
PPy	PCL	PCL electrospun with Py and then polymerized with oxidant, then electrospun	9:1 CHCl_3_:DMF	20 kV	1 mL/h	13 cm	200–500 nm	8.14 × 10^−7^–15.60 × 10^−7^ S/cm (4-point probe)	Contact angle: 93–103°, Young’s modulus: 8.50–10.50 MPa, elongation at break: 320.07–540.05%	Bone tissue engineering	[251]	-
PPy	PCL	PCL electrospun then immersed in Py and oxidant containing baths, then PCL core removed with DCM	TFE	17 kV	1 mL/h	14 cm	598 ± 213 nm to 763 ± 327 nm	-	-	Photothermal therapy	[252]	Hollow shell
PANI	PCL	PANi incorporated into PVA hydrogel and then cast onto PCL	3:1 CHCl_3_:MeOH	10 kV	0.5 mL/h	10 cm	-	3.23 × 10^−2^ S/cm (max, 4-point probe)	Contact angle: 27.63 ± 0.35° to 58.60 ± 0.53°, Young’s modulus: 49.70 ± 0.42 to 52.15 ± 0.41 MPa, elongation at break: 14.6 ± 0.23% to 15.3 ± 0.21%	-	[253]	-
PEDOT:PSS	PCL	Dopamine-modified electrospun PCL dip-coated in PEDOT:PSS	15:85 EtOH:DCM	1 kV	2.5 mL/min	-	-	5 × 10^4^ to 17 × 10^6^ Ω/sq (4-point probe)	Contact angle: 0°	Muscle regeneration	[254]	-
PANI	PCL	Electrospun PCL/osteogenon/gelatin/calcium nanoparticle composite printed with PANI using inkjet printer	1:1 CHCl_3_:MeOH	25 kV	1.5 mL/h	20 cm	2000–4000 nm	10^−3^ S/cm (electrochemical impedance spectroscopy)	-	Bone tissue engineering	[255]	-
PPy	PLGA	In situ polymerization of PPy on PLGA mat	Trifluoroethanol (TFE)	20 kV	0.6 mL/h	20 cm	936 ± 412 nm (about 150 nm thick layer)	0.118 S/cm (4-point probe)	-	Peripheral nerve regeneration	[256]	Conductivity of PPy film: 0.302 S/cm, conductivity of PPy/PLGA film: 5.36 × 10^−3^ S/cm
MEH-PPV, PEDOT:PSS	PVP	TiO_2_/PVP electrospun, then PVP removed by calcination. MEH-PPV then spin-coated. PEDOT:PSS was then deposited	EtOH	14 kV	-	8 cm	~200 nm thick MEH-PPV and 70 nm thick PEDOT:PSS	-	-	Photovoltaics	[257]	-
MEH-PPV, P3HT	PVP	Blend of MEH-PPV and PCBM electrospun with PVP. P3HT/PCBM added with spin coating	CHCl_3_	8 kV	0.5 mL/h	15 cm	300–450 nm	1.02–1.34 × 10^−7^ S/m (tunneling AFM)	-	Solar cells	[210]	Blend, core/shell
PEDOT	PVA	Blend of PVA and graphene oxide electrospun, then PEDOT electrochemically polymerized on surface	H_2_O	15 kV	1.2 mL/h	15 cm	Globular porous structure	-	224.27 F/g (specific capacitance)	Supercapacitors	[258]	PEDOT film specific capacitance: 167.92 F/g, PEDOT/PVA nanofiber specific capacitance: 182.73 F/g
PANI	PMMA	In situ polymerization	THF	7 kV	-	6 cm	2500–3500 nm, PANI nanoparticles	-	-	Ammonia sensor	[259]	-
Polyquinoxaline-based	PVA	PVA electrospun then modified with amine and dip coated in polyquinoxaline solution	1.11:3:7 Tetraethoxysilane:EtOH:water	20 kV	0.5 mL/h	10 cm	360 ± 90 nm (before dip coating)	-	-	Organophosphorous compound detection	[260]	-
PANI	PMMA	PMMA electrospun, then in situ polymerization of aniline and immobilization of Au nanoparticles	7:3 DMF:acetone	30 kV	1 mL/h	20 cm	400–500 nm	50 ± 15 S/m	-	O_2_ radical sensing with immobilized enzyme	[261]	-
PEDOT:PSS	PLLA	PLLA electrospun and then EDOT electrochemically polymerized	CHCl_3_	-	0.25 mL/h	10 cm	110 ± 8 nm (30 ± 8 nm PEDOT coating)	19.3 ± 5 MΩ-s (impedance)	-	Glucose detector	[262]	-
PPy	PAN	PAN electrospun with oxidant and then exposed to Py vapors	DMF	8–22 kV	1–3 mL/h	9–23 cm	650 ± 10 nm (PAN)	-	-	Glucose detector	[263]	-
poly-4-(4,7-di(thiophen-2-yl)-1H-benzo[d]imidazol-2-yl)benzaldehyde (PBIBA)	Nylon 6,6	Nylon, MWCNT mixtures electrospun then PBIBA polymerized electrochemically	Formic acid	15 kV	1.0 mL/h	10 cm	70 ± 20 nm (nylon/MWCNT)	-	Contact angle: 66.65 ± 0.76° to 67.81 ± 2.57°	Glucose detector	[264]	-
PEDOT	NBR/PEGDM	NBR/PEGDM electrospun then exposed to EDOT vapor, then immersed in oxidant	CHCl_3_	14 kV	1.5 mL/h	10 cm	6260 ± 2990 nm	6 ± 3 S/cm	-	Non-Hodgkin lymphoma gene detector	[265]	-
PPy	PVP	PVP electrospun, then in situ vapor phase polymerization of PPy with Au nanoparticles	DMF	20 kV	0.8 mL/h	18 cm	100 nm (PPy capsules), 20 nm (wall thickness)	5.3 × 10^−5^ S/cm to 8.5 × 10^−3^ S/cm (4-point probe)	-	Wearable NH_3_ sensor	[266]	-
PANI	PVDF	In situ chemical oxidative polymerization of PANI on electrospun PVDF	1:1 DMF:acetone	10 kV	-	8 cm	-	Retained until ultimate strain	Elongation at break: 110.53%	Strain sensor	[267]	-
PPy	PLA-Silk Fibroin-Collagen	In situ chemical oxidative polymerization of PANI on electrospun PVDF	HFIP	20 kV	0.5 mL/h	20 cm	122 ± 28 nm, PPy coating amorphous	-	-	Motion, respiration sensor	[268]	-
PEDOT:PSS	Poly (vinylidene fluoride-co-hexafluoropropene)	In situ vapor oxidative polymerization of PANI on electrospun PVDF-oxidant collected on PEDOT:PSS/PET substrates	1:1 DMF:THF	10.5 kV	6–8 μL/min	3–12 cm	480 ± 34 nm	7 × 10^4^ Ω/sq (4-point probe)	-	Wearable pressure sensor	[269]	-
PPy	PAN	Double-conjugate electrospinning then in situ chemical oxidative polymerization	DMF	-	-	-	10–70 nm coating	10.5 S/cm	-	-	[270]	Conductivity and elongation at break increase to 94.37 S/cm and 40%, respectively, with the addition of graphene
PPy	PAN	PAN electrospun then PPy solution dripped on mats	DMF	16 kV	0.65–0.8 mL/h	20 cm	-	-	-	Photocatalytic decontamination of water	[271]	-
PEDOT:PSS	PMMA-TiO_2_	PMMA electrospun with TiO_2_ precursor then calcined, then immersed in oxidant, then vapor phase polymerization of PEDOT	1:1 CHCl_3_:DMF	25 kV	0.5 mL/min	11 cm	269–412 nm	-	-	Photocatalytic decontamination of water	[272]	-
PANI	PS	PS mat in situ chemical oxidative polymerization with PANI	DMF	17 kV	0.5 mL/h	15 cm	1600 ± 400 nm (PS)	0.016 S/cm	Contact angle: 106 ± 2°	Adsorption of heavy metal ions	[273]	-
PANI	PAN	In situ chemical oxidative polymerization	DMF	20 kV	1 mL/h	10 cm	333 nm	-	Contact angle: ~150°, Young’s modulus: 302.8 ± 78.30 MPa, elongation at break: 47 ± 1%	Oil/water emulsion separation	[233]	Electrospun PANI/PAN blend (10–40 wt.% PANI) has Young’s modulus of 12.64 ± 2.91 MPa
PANI	PU	In situ chemical oxidative polymerization	DMF	20 kV	0.1 mL/h	12 cm	155–270 nm (average 207 nm)	-	-	Opium alkaloid detection	[274]	-
PANI	Polycaprolactam	In situ chemical oxidative polymerization	Formic acid	“+10 kV to −5 kV”	0.5 mL/h	15 cm	190–270 nm	-	-	Drug detection in human plasma	[275]	-
PEDOT:PSS	PU	Electrospun MWCNT/lauric Acid/PU dip coated in PEDOT:PSS	DMF	12 kV	0.8 mL/h	25 cm	200–300 nm	13.3 to 39.7 S/cm (sheet resistance)	Elongation at break: 200–650%	Stretchable conductor	[276]	-
PEDOT:PSS	PVA	Blend electrospun then dip coated then coated with AgNPs	DMSO	23 kV	0.4 mL/h	9 cm	440 nm	0.67 to 41.5 S/cm (4-point probe)	Young’s modulus: 1.34–7.47 MPa, elongation at break: 2.99–9.93%	Flexible thermoelectric generator	[236]	Combination of blend (5 wt.% PEDOT in PVA) and core/shell
PPy	PLA	PLA electrospun then immersed in oxidant then vapor phase polymerization	CHCl_3_	15–25 kV	1.5–2.5 mL/h	15 cm	7620 ± 0.13 to 9300 ± 0.15 nm	Up to 0.5 S/cm (4-point probe)	-	Shape memory device	[277]	-

## 5. Applications

Electrospinning allows a wide range of polymers to be used to form nanofibrous structures with high surface-to-volume ratios, porosity, and orientation-dependent properties. The methods discussed above have enabled the use of electrospun CPs in applications, from biological to wearable materials. The addition of CPs introduces tunable physical and electronic properties with the application of an electrochemical stimulus. One important consequence of this is that when CPs are electrospun by themselves or co-electrospun with another polymer, the higher conductivity of the polymer solution often results in the formation of thinner fibers [251].

### 5.1. Bioomedical Applications

Electrospun nanofibers can be functionalized with bioactive molecules such as drugs and proteins. The addition of CPs is especially suitable for application to biological processes which are enhanced by the application of a current or heat.

#### 5.1.1. Wound Healing and Therapy

**Figure 15:**
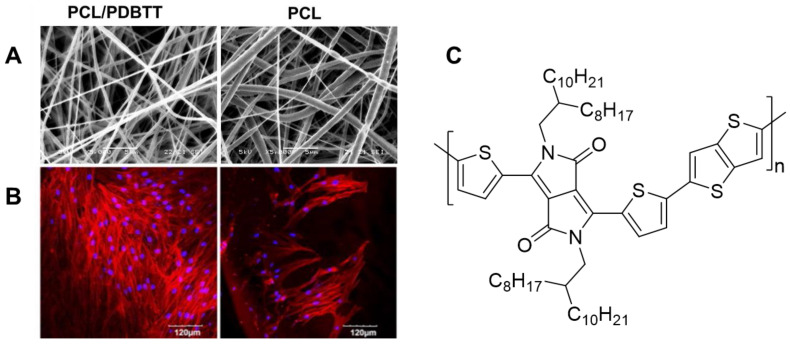
Wound healing application of electrospun CPs [204]: (**A**) SEM images of electrospun PCL/PDBTT and PCL nanofibers; (**B**) confocal and SEM images of extent of growth of HDF on electrospun PCL/PDBTT and PCL nanofibers, showing enhanced HDF growth on PCL/PDBTT with light stimulation; (**C**) structure of thiophene/pyrrole-basedpolymer PDBTT. Reprinted with permission from Ref. [204]. Copyright 2017, with permission from Elsevier.

Human dermal fibroblast (HDF) growth on electrospun nanofibers comprising PCL and a thiophene/pyrrole-based polymer (PDBTT) at a ratio of 15:1 were shown (Figure 15) to be enhanced due to the ability of the nanofiber mat to conduct a photocurrent after absorption in the near-infrared region (NIR) [204]. This was due to the promotion of cellular processes and division as a result of the electric and magnetic fields induced by the photocurrent. Similar wound-healing properties were also observed by another group on rat wounds covered with membranes of electrospun PANI-co-poly(o-aminobenzenesulfonic acid)/poly(vinyl alcohol)/chitosan oligosaccharide, with almost complete healing after 15 days [278].

The ability of CPs to absorb in the NIR due to conjugation has also led to photothermal therapeutic applications. Hollow fibers of PPy were prepared via in situ polymerization of pyrrole on PCL electrospun mats followed by dissolution of the substrate PCL [252]. These fibers, along with polydopamine-coated mats of PCL [279], were shown to be effective in the photothermal killing of model cancer cells, with the fibrous structure assisting in the continuous heat flow all over the treated cells and the high surface area of the mat resulting in increased heating effects. In addition, these structures, as well as a PANI-coated electrospun PCL mat, were shown [253] to have a tunable binding interaction with the chemotherapeutic doxorubicin; the membranes exhibited high swelling capabilities and reduced hydrophilicity, indicating their suitability for drug delivery and localized treatment applications.

#### 5.1.2. Tissue Scaffolds

The conducting polymers PPy, PTh, and PANI have shown good biocompatibility, as they have been found to be noncytotoxic [205,254,255]. Porous, aligned electrospun mats containing these polymers mimic the structure of the human extracellular matrix. This structure allows for the attachment of proteins and cells, leading to applications as scaffolds for tissue engineering. In addition, the enhanced conductivity of CP-containing oriented fibrous scaffolds allows for better communication between cells, enhancing tissue growth. This is particularly of interest in tissues in which the conduction of impulses or signals are important, such as in nerve, muscle, and bone tissue. The main issue faced in these types of applications is the weak mechanical properties of scaffolds made up entirely of CPs, which tend to have low molecular weights and poor mechanical properties, such as brittleness. These are remedied by the incorporation of biocompatible high-molecular-weight insulating polymers such as PLGA, PEO, PCL, and poly(vinylidene difluoride (PVDF) [206].

**Figure 16:**
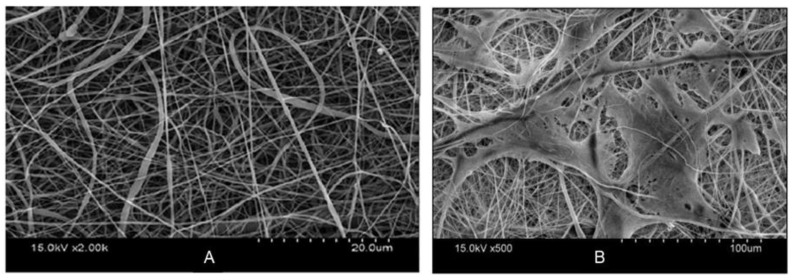
SEM images showing electrospun PVDF/PANI blends [207] (**A**) before and (**B**) after 3-day culture of MC3T3 cells. Reprinted with permission from Ref. [207]. Copyright 2020, John Wiley & Sons.

Bone tissue scaffolds were synthesized from PANI deposited on electrospun PCL/osteogenon/gelatin/calcium phosphate nanoparticles [255] and electrospun mats of PANI/PVDF and PPy/PVDF [207]. Importantly, the porous structure of CP-containing scaffolds allows for the implantation of calcium phosphate nanoparticles and drugs in addition to promoting cell growth and enhancing intercellular communication through enhanced conductivity; growth of MC3T3 cells on the scaffold is readily apparent when comparing SEM images from before and after cell culture (Figure 16) The CPs in these scaffolds also mimic the piezoelectric property of natural bone cells, which allows for better cell–scaffold communication [251].

**Figure 17:**
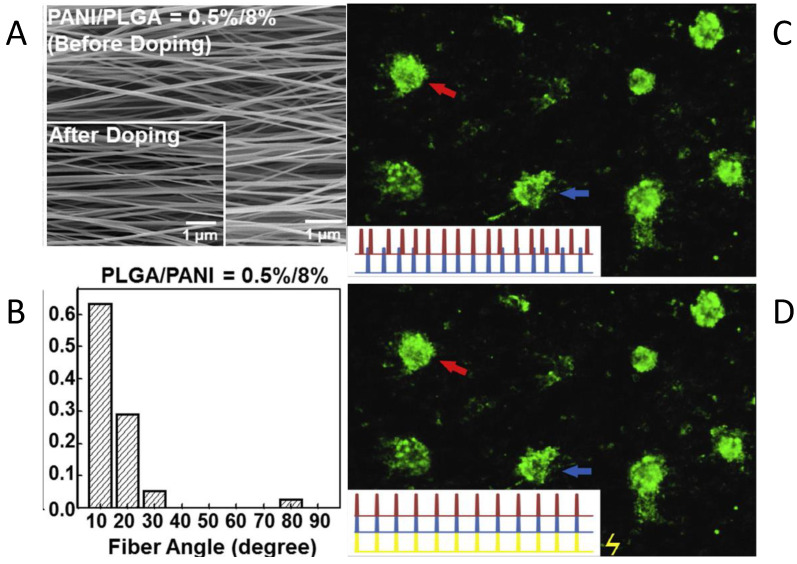
Cardiac tissue scaffold made of a PANI/PLGA blend [208]. (**A**) SEM of PANI/PLGA nanofibers before and after doping with HCl. (**B**) Histogram of fiber angle distribution of PANI/PLGA showing a high degree of alignment. (**C**) Beating frequencies of indicated red and blue cell clusters without electrical stimulation. (**D**) Same beating frequencies as (**C**) but with electrical stimulation applied (yellow) showing synchronization of beating between electrical stimulation and beating in the red and blue cell clusters. Reprinted with permission from Ref. [208]. Copyright 2013, with permission from Elsevier.

In the field of cardiac and muscle tissue engineering, the properties of CP-containing scaffolds that have emerged as important are the presence of charges on doped CPs, their fibrous structure, and their conductivity. Scaffolds of PANI/PLGA, PANI/PCL and PEDOT:PSS/polydopamine/PCL blends [208] have been used in this regard. These scaffolds allow for the adhesion of growth factors, stimulated controlled cardiac heating, and not only the attachment and proliferation of cells but also successful elongation [209] and differentiation [254]. As can be seen in Figure 17A,B, a high degree of alignment was attained for PANI/PLGA nanofibers; increasing PLGA content increased alignment. Doped scaffolds, which were expected to have increased conductivity relative to neutral scaffolds, promoted adhesion of cardiomyocytes, which associated with each other to form isolated cell clusters (Figure 17C). The cardiomyocytes in each cluster beat synchronously, and application of an electric field caused the beating of all the clusters to synchronize when higher amounts of PANI were used (Figure 17D).

**Figure 18:**
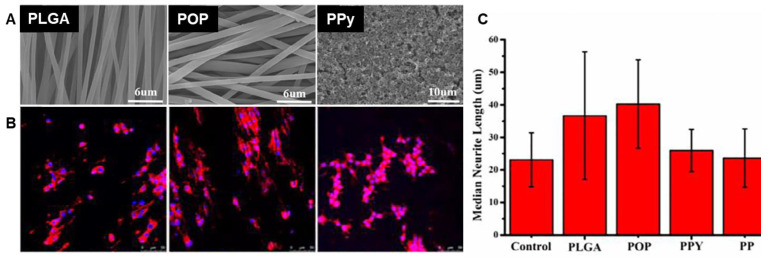
Nerve tissue scaffold application of electrospun CPs [256]. Left to right: electrospun PLGA, PPy-coated electrospun PLGA (labeled POP), and PPy film. (**A**) SEM images. (**B**) Laser scanning confocal microscopy images of PC12 cells grown on the electrospun and cast scaffolds, showing improved neurite proliferation and directional growth in PPy-coated electrospun PLGA. (**C**) Comparison of the median neurite lengths of PC12 cells grown on polymer scaffolds, showing the best neurite growth for PPy-coated electrospun PLGA (labeled POP). Reprinted with permission from Ref. [256], Copyright 2018, with permission from Elsevier.

Lastly, because of the central function of the nervous system in transmitting impulses from one part of the body to another, nerve growth applications have received considerable attention in the field of CP-based tissue scaffolds. PPy-coated electrospun PLGA and P3HT/poly(N-vinylpyrrole) [256] scaffolds (Figure 18) have been used for this application. The alignment of the fibers in the scaffold enables the oriented growth of nerves (Figure 18B), and electrical stimulation mimics the electrophysiological environment of native nerve cells and promotes their growth and proliferation; longer neurites are obtained from PPy-coated nanofibers (Figure 18C) [256]. 

### 5.2. Electronic Devices

The enhanced charge transport properties as well as uniformity in morphology of electrospun nanofibrous CPs lead to their applications in the field of electronic materials, particularly in the fabrication of energy conversion, energy storage, and other electronic devices. 

#### 5.2.1. Photovoltaics

The conjugation present in CPs enables them to absorb photons from the UV, visible, and infrared regions [206]. The uniform nanofibrous structure produced by electrospinning polymer solutions properly prevents the formation of agglomerates which serve to concentrate photon absorption. Moreover, the electrospinning process extends the chains of the polymers being electrospun, resulting in an increase in conjugation length in the polymer and an increase in the efficiency of optical absorption [199]. 

The increased surface area, porosity, and tunability of nanofibrous structures over non-electrospun systems also lends devices made from these structures many advantages in the field of energy conversion. The photons absorbed by CPs create pairs of electrons and positively charged “holes” in the system [280]. Increasing the surface area of materials with CPs has the effect of allowing enhanced photon absorption, complete charge separation, and easy doping of CPs. Incorporating electron acceptor materials such as TiO_2_ into the device separates these electron–hole pairs, with the CPs serving the purpose of being the hole-transport layer. The increased porosity of nanofibrous structures allows for the incorporation of these nanoscale electron-acceptor materials, or vice versa [257].

A recent report [206] postulated the potential of CP nanofiber blends for use in photovoltaic devices: PANI, PPy, and PTh were each blended at 1% and 3% CP content with PAN and then electrospun into nanofibers. The incorporation of CPs into the fibers decreased average fiber diameters by as much as 43%, possibly due to the increased solution conductivity during electrospinning. Even at such low CP concentrations, the nanofibers exhibited reduced bandgaps and improved absorption and light refraction relative to bare PAN. 

A hybrid solar cell made from a nanowire device of PEDOT:PSS-coated MEH-PPV layer on electrospun TiO_2_ was found to have enhanced power conversion efficiency and charge conduction [257]. This structure also allows for the incorporation of many other nanoparticles for varied applications, such as in the case of a photovoltaic cell device based on co-electrospun silver nanoparticles in ethyl glycol and a PFO-based polymer, with the silver nanoparticles also acting as a solar concentrator [200]. Other examples include incorporation of a fullerene-containing polymer into nanofibers with P3HT and poly(vinylpyrrolidone) [210] and another application involving quantum dots [199]. The alignment of nanofibers in these kinds of devices also plays an important role in their function. Aligned structures enhance the collection and transport of charges, leading to an increase in the number of junctions between hole-transporting CPs and electron accepting nanoparticles, which further increases charge collection overall which can flow in ordered conductive pathways. 

#### 5.2.2. Capacitors

Nanofibrous, high-surface area CP-based materials can store charge through two mechanisms: through pseudo-capacitance, which is the capacity of the structure to undergo repeated reversible redox reactions, and electrical double-layer capacitance, which is the capacity of the structure to promote accumulation of charges, separate these charges, and allow for ion transport [258]. CP-based electrochemical capacitors take advantage of both these charge storage mechanisms to deliver higher power and higher energy storage per unit mass than batteries. PEDOT-coated electrospun poly(vinyl alcohol) (PVA)/graphene oxide nanofibers and PEDOT:PSS/PEO [211,258], PANI/carbon nanotubes/PEO and PANI/PE [212,213], and polyindole/carbon nanotube [214] composite materials have been used as collectors and electrodes in this regard. The high surface areas of the fibers lead to improved capacities relative to films of the same polymers, while the flexibility of the fibers may lead to conformal energy storage.

Fotia and coworkers attempted to electrospin neat camphorsulfonic acid-doped PANI but were only able to obtain electrosprayed PANI mats [281]. Instead, the electrospun nanofibers of blends of doped PANI with carrier polymers poly(methyl methacrylate) (PMMA) and poly(vinyl acetate) (PVAc) had between 50 and 75 wt.% PANI in the blends. Conductivities of these fibers were significantly lower than the electrosprayed pure PANI mats (10^−2^ S·cm^−1^), which were much lower than doped PANI films (600 S·cm^−1^). The conductivity of the PANI/PMMA blended nanofibers (10^−7^ S·cm^−1^) was two-orders-of-magnitude lower than that of the PANI/PVAc blends (10^−5^ S·cm^−1^), with only minimal differences found with changes in the weight percentage of PANI. The authors postulated that this was due to the much lower conductivity of pristine PMMA (10^−10^ S·cm^−1^) relative to that of pristine PVAc (10^−6^ S·cm^−1^) and to lower compatibility of PANI with PMMA. Specific capacitances of these nanofiber composites were relatively low, between 0.2 and 0.4 F·g^−1^ at 10 mV·s^−1^. The team attempted to improve performance with addition of graphene oxide or iron oxide; however, oxide addition did not provide a significant improvement in specific capacitance and decreased conductivity, possibly due to aggregation of the oxides.

One approach to improving the capacitive performance of CP-based capacitors is by addition of conductive carbonaceous materials such as carbon nanotubes or graphene oxide. In this way, devices combine the pseudocapacitive behavior of the CP with electrical double-layer capacitive behavior of the carbonaceous materials [282,283]. Electrochemical capacitors were prepared by electrospinning poly(thioaniline) with PVA, with and without addition of graphene oxide [282]. Addition of graphene oxide increased capacitance from a respectable 116 F·g^−1^ at 0.6 A·g^−1^ to 166 F·g^−1^. Addition of graphene oxide also increased cycle stability, from a 32% decrease after 5000 cycles without graphene oxide to only a 10% decrease when graphene oxide was added.

#### 5.2.3. Field-Effect Transistors and Diodes

Near-one-dimensional field effect transistors (FETs) made from CPs could enable increased component density in circuits; fabrication of these devices from electrospun CPs is potentially simpler than using traditional lithographic techniques [191]. An FET was successfully constructed from co-electrospun P3HT and PMMA on SiO_2_; the oriented, conductive nanofibrous structures of these P3HT-based nanofibers have been used as field-effect transistors [191]. 

Lee and coworkers prepared P3HT-based FETs via coaxial electrospinning of P3HT [198]. Neat P3HT nanofibers prepared via coaxial electrospinning of P3HT/CHCl_3_ in the inner nozzle and chloroform in the outer nozzle demonstrated a field-effect mobility of 0.017 cm^2^·V^−1^·s^−1^ and an on/off ratio of 100. The group also prepared P3HT/PCL blends with P3HT content ranging from 50% to 90% by weight [198]. Nanofibers electrospun from the P3HT/PCL blends were tested for use in FETs; as PCL content increased, the field effect mobility decreased from 0.017 cm^2^·V^−1^·s^−1^ for 100% P3HT to 0.00047 cm^2^·V^−1^·s^−1^ for 50% P3HT: a 97% reduction. The authors hypothesized that, as PCL content increases, phase separation occurs between the P3HT and PCL domains, allowing the PCL domains to act as defect sites and degrading hole mobility.

**Figure 19:**
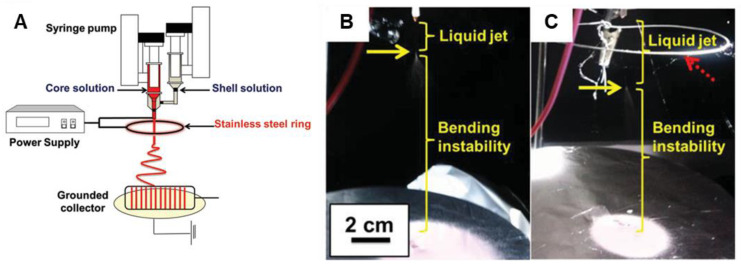
Use of a secondary electric field below the syringe (**A**) results in prolonged liquid jets (**B**,**C**) that enhance charge transport, resulting in dramatically enhanced mobility [200]. Reprinted with permission from Ref. [200]. Copyright 2015, John Wiley & Sons.

Chen and coworkers [200] found that adding a second electric field introduced additional extensional force that prolonged the liquid jet by 3–5 cm (Figure 19). This modified procedure produced thinner fibers (reduced from 2.11 to 1.39 µm) of highly extended and oriented P3HT/PMMA core/shell nanofibers. The PMMA shell could then be removed by solvent etching, producing 144 nm diameter P3HT nanofibers vs. 414 nm without the secondary electric field. The strong extensional forces provided by the secondary electric field apparently caused the P3HT chains to align along the jet, dramatically enhancing charge carrier mobility in FETs by more than three orders of magnitude to 1.62 × 10^−1^ cm^2^·V^−1^·s^−1^.

P3HT/poly(lactic acid) (PLA) has also been used on n-doped Si/SiO_2_ in order to form highly efficient diodes [215]. Interestingly, while neither P3HT or PLA electrospins well alone, blends of the two polymers produced long composite fibers. These fibers were deposited onto prepatterned gold electrodes on n-doped Si/SiO_2_ substrates to form versatile p–n diodes that could be tuned either optically or electrostatically. 

### 5.3. Sensing

Another important application of electrospun CPs lies in the field of sensors for gases, ions, and even biological molecules. These rely on (a) secondary interactions between the analytes and the substrates, (b) the ability of the analytes to reduce/oxidize the substrates, or (c) the change in the electrochemical potential induced in the substrate by the analyte binding to a sensor immobilized on the substrate [284]. The sensing ability is measured as a function of changing conductivity/resistivity of the substrate or changing absorption or fluorescence of the substrate [45]. The high surface area of nanofibers improves sensing ability over thin films [131]. The nanofibrous structure of electrospun CPs has the added advantage of increasing the number of conduction pathways in the sensing material, lending it added sensitivity and faster response rate [285]. For a thorough discussion on the use of electrospun nanofibers as sensors, see the review article by Halicka and Cabaj [286].

#### 5.3.1. Gas and Humidity Sensors 

Gases ranging from common potentially harmful or pollutant gases such as ammonia, volatile organic compounds, ethanol, liquified petroleum gas, organic solvents, and ethanol to warfare agent gases such as organophosphorus nerve agents, nitroaromatics, and hydrogen gas have been detected on sensors based on electrospun CPs. Ammonia [216,259], hydrogen [217], and liquified petroleum gas [209] have been found to exchange electrons with PANI and increase the electrical resistance of PANI/ZnO composites, while the same mechanism has been found operative in the detection of electron-accepting nitroaromatics [218] such as trinitrotoluene by electron-donating polyfluorene (PFO)-based materials in composites with PS and gelatin. Volatile organic compounds such as benzene and toluene have been demonstrated to swell nanofiber composites of PEDOT:PSS/carbon nanotubes/poly(vinylpyrrolidone) [219], PANI/isoindigo-based copolymer/PMMA [220], and PFO/PMMA [221], destroying conduction pathways in these materials and increasing electrical resistance. Ethanol has been detected with a sensor made from SnO_4_/PANI/poly(hydroxy-3-butyrate), with hydrogen bonding between ethanol and amine domains, as well as dopants, being the operating mechanism [222]. Lastly, the fluorescence of conjugated CPs like polyquinoxaline and poly(phenylene vinylene) (PPV) has been shown to be quenched by π–π interactions with organophosphorous nerve gas [260] and aromatic organic solvents [223].

Humidity sensors are widely implemented in applications including pharmaceuticals, paper manufacturing, electronics, textiles, packaging, and food [287]. Many types of organic materials have been used for resistive and capacitive humidity sensors [288], and CP nanofibers are only a small subset of the approaches used. Naval Research Laboratory scientists trying to develop CP-coated nanofiber-based chemical warfare agent sensors discovered that humidity significantly impacted the conductivity of the fibers [289]. Since then, many researchers have explored the use of CP nanofibers for humidity sensing. Aussawasathien and coworkers [224] noted that, when designing humidity sensors, it is important to ensure that the polymer nanofibers do not swell or dissolve in water. Thus, Li and coworkers prepared a thermally crosslinkable PANI-containing nanocomposite that was electrospun, crosslinked, and tested for humidity sensing [225]. While the sensors were effective at detecting high humidity levels in the absence of PANI, addition of PANI decreased baseline impedance, allowing lower detection limits. They found a two-orders-of-magnitude change in impedance when relative humidity changed from 22% to 97% and stated that the high surface area increased ability to rapidly absorb and desorb water, leading to more rapid detection. 

#### 5.3.2. Ion Sensors

Electrospun CP-based sensors have been used to detect a wide variety of ions, including phosphate, mercury (II), and superoxide. Electrochemical detection of phosphate was accomplished using PAN/PT composite nanofibers deposited onto indium tin oxide-coated glass [226]. As phosphate concentration increased, the oxidation potential of PT in the fibers decreased. Peak current response was found to increase as the PT content in the fibers increased.

**Figure 20:**
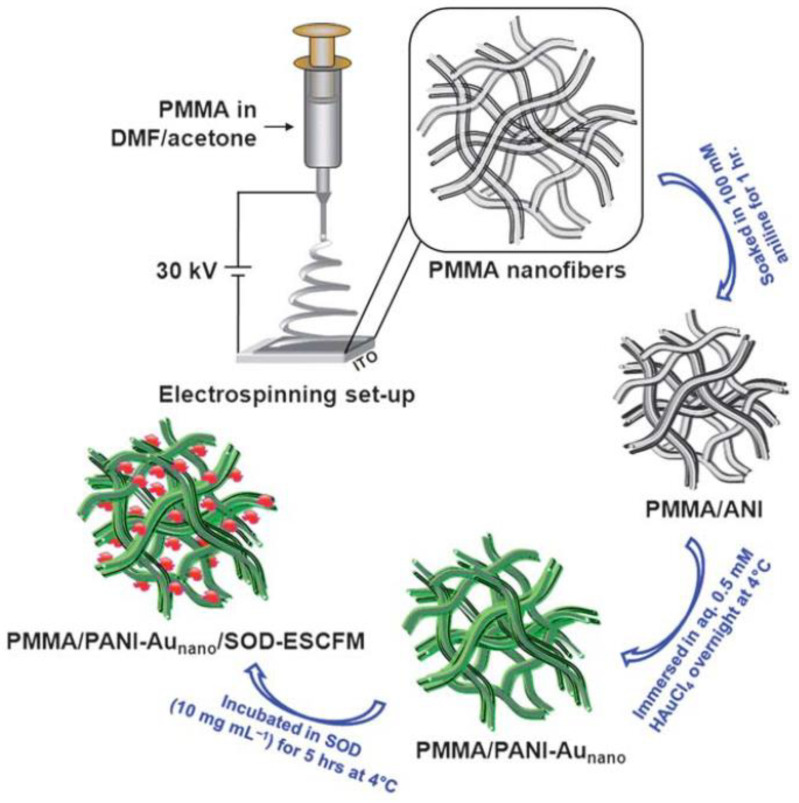
PMMA/PANI superoxide sensors were prepared by electrospinning PMMA and coating the resultant nanofibers with PANI via chemical oxidation of aniline with hydrogen tetrachloroaurate [261]. The enzyme superoxide dismutase was then bonded to the gold nanoparticles formed in the PANI during oxidation, and the enzyme was used to detect superoxide. Republished with permission of Ref. [261]; permission conveyed through Copyright Clearance Center, Inc.

High levels of the superoxide anion (O_2_^−^) can result in inhibition of cell growth, mutagenesis, and cell death [290]. Santhosh and coworkers electropun PMMA nanofibers and oxidatively deposited a PANI coating on the surface (Figure 20). By utilizing hydrogen tetrachloroaurate as the oxidant, they were able to form gold nanoparticles in the PANI. The enzyme superoxide dismutase was then immobilized on the gold nanoparticles. The resultant composite could then be used as amperometric sensors for superoxide (O_2_^.−^) [261]. The sensors had good detection limits (0.3 µM), rapid response times (4 s), and good stability and reproducibility.

A colorimetric sensor was developed using PANI/polyvinylbutyral/poly(amide-6) nanofibers for the detection of Hg(II). The interaction between mercury and PANI, a multistep process involving complexation of Hg^2+^ to aniline nitrogens, translates to color changing for rapid visual evidence of mercury exposure (Figure 20) [227]. The color change in the sensors could be observed with the naked eye, or colorimetric responses could be quantitatively converted to numerical values. 

#### 5.3.3. Biosensors

**Figure 21:**
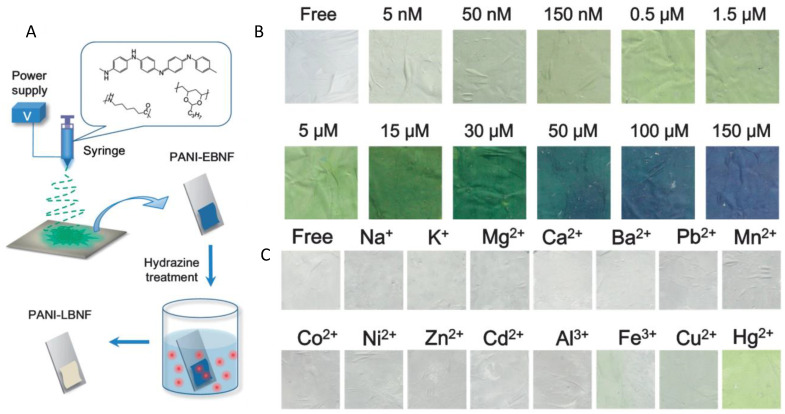
PANI-containing electrospun composite used for colorimetric sensing of Hg(II): (**A**) process for synthesis of PANI sensor strip showing electrospinning of a mixture of PANI, polyvinylbutyral, and poly(amide-6) and then treatment with hydrazine; (**B**) result of incubating PANI sensor strip in Hg(II) aqueous solutions showing progressive discoloration and darkening after contact with increasing Hg(II) concentration; (**C**) sensor strip after incubation in solutions of different metals, showing selectivity of sensing to Hg(II). Republished with permission of The Royal Society of Chemistry from [227]; permission conveyed through Copyright Clearance Center, Inc.

The ability of CPs to be modified with a wide range of bioactive species has led to extensive development of CP biosensors [45], Sensors for the detection of additives, antigens, and biomolecules have also been developed based on electrospun CPs. These work by either quenching interactions with analytes or by changing electrochemical potential as a result of binding of analyte on another sensor immobilized on the surface of the material. Sudan dyes, which are illegal food additives, have been found to quench the fluorescence of a PPV/PVA composite [228]. Antibodies to immunoglobulin G, a measure of immunity, and carcinoembryonic antigens, have been immobilized on PPy/PEO [229] and PEDOT:PSS/PVA nanofibers (Figure 21) [230], respectively. Enzymes for the detection and possible degradation of glucose, such as glucose oxidase, have been immobilized on the surface of PEDOT/poly(L-lactide) [262], PAN/PPy [263], and poly-4-(4,7-di(thiophen-2-yl)-1H-benzo[d]imidazol-2-yl) benzaldehyde)/multiwalled carbon nanotubes/nylon 6,6 fibers [264]. A sensor for the detection of non-Hodgkin’s lymphoma has even been developed, based on an oligonucleotide immobilized on nitrile butadiene rubber/poly[(ethylene glycol)dimethacrylate]/PEDOT [265].

#### 5.3.4. Wearable Sensors

Sensors that can be worn on the human body have also been developed, for applications including the detection of harmful substances in an area [266], analysis of body fluids [201], monitoring of vital signs [268,269,270], and recording of motion [268]. Promising sensors are those that have rapid response times to external stimuli and are stable through continued use and cycling between loading and unloading of the stimulus [291]. As with other sensors, the wearable chemical sensors operate on the principle of the CP substrate undergoing electron transfer with the analyte in question [231,285]. Mechanical wearable sensors operate on the principle of piezoelectricity/piezoresistivity: the application of an external pressure to layers of CP brings various regions of the material in contact with each other [231,292,293]. An increase in the number of contacting regions as a result of an increase in pressure induces an increase in the conductivity of the material, which is then interpreted to extrapolate a pressure or frequency reading after calibration. The nanofibrous structure imparted by electrospinning, with large porosity and surface area, imparts greater sensitivity and faster reaction time of the sensor to the measurement of the analyte or behavior [292]. 

**Figure 22:**
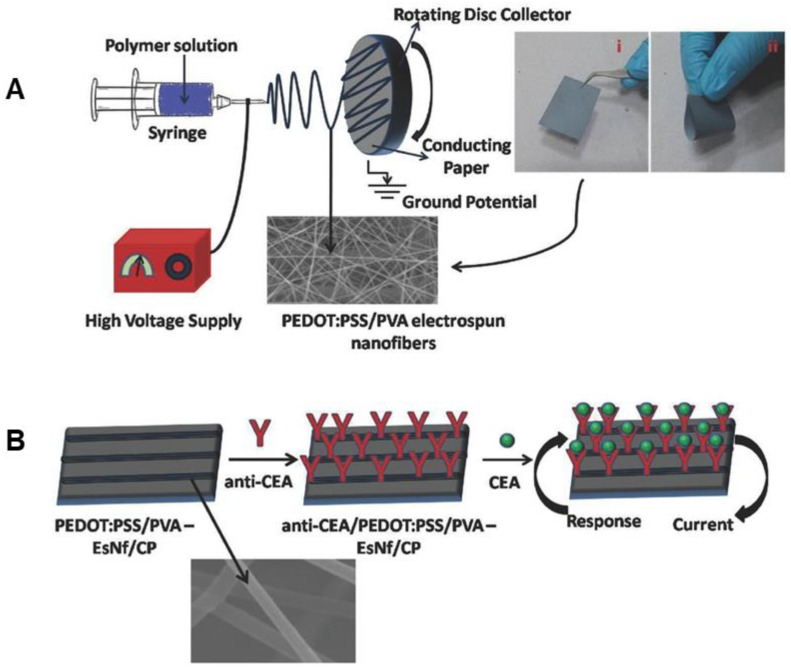
Biosensor developed from PEDOT:PSS/PVA to detect carcinoembryonic antigen (CEA) [230]: (**A**) diagram of electrospinning setup of PEDOT:PSS/PVA nanofibers; (**B**) schematic diagram of PEDOT:PSS/PVA-electrospun nanofibers/conducting paper and immobilization of CEA antigen. Reprinted from [230]. Copyright 2016, John Wiley & Sons.

**Figure 23:**
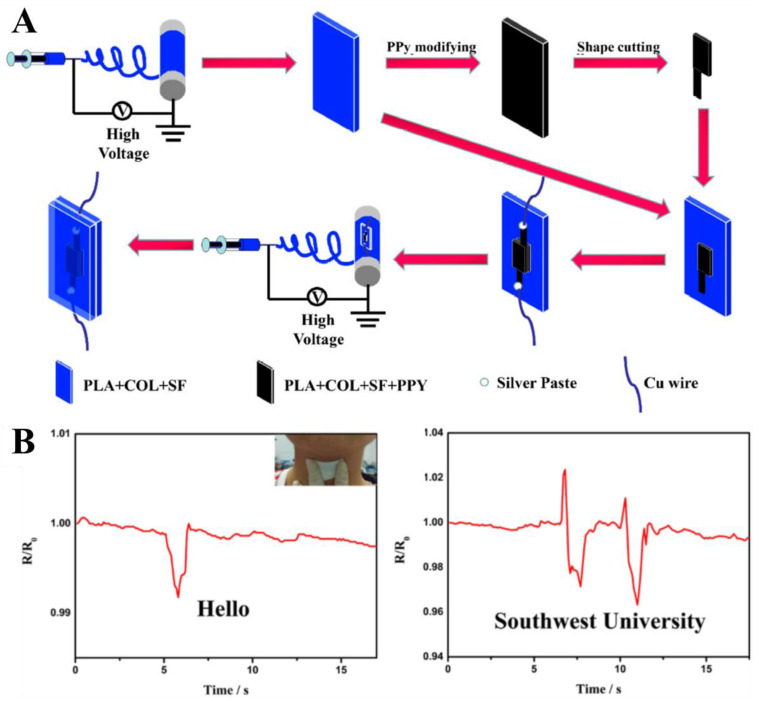
Human motions detectable by a piezoresistive sensing device composed of PPy-coated PLA/silk fibroin/collagen [268]: (**A**) fabrication via PPy coating of electrospun PLA/silk fibroin-collagen nanofibers; (**B**) vocal cord vibrations detected by a change in resistance in the sensor. Reprinted from [268], available at https://doi.org/10.3390/polym10060575, accessed on 1 December 2022.

An ammonia detector was constructed out of in situ polymerized PPy on sintered electrospun PVP/vanadyl acetylacetonate/gold(III) chloride trihydrate [266]. Sweat pH was measured using coaxial electrospun blended PANI and polyurethane (PU) [201]. PEO/PEDOT:PSS [231] and PANI/PVDF composites [267] were used as strain sensors in order to detect the flexion of the wrist, and movement and tapping of the finger. Zhao and coworkers prepared a wearable piezoelectric sensor using three electrospun polymer layers (Figure 22A) [268]. They electrospun nanofibers from a blend of PLA, silk fibroin, and collagen. They then used oxidative polymerization to coat some of the nanofibers with PPy. These were then used to prepare a composite device using the PLA/silk fibroin/collagen nanofiber mat as the outer layer and the PPy-coated mat as the inner layer. The resultant flexible, wearable piezoelectric sensor was able to detect minute variations of pressure in the body and environment such as pulse, respiration rate, and throat vibrations (Figure 23B) [268]. Similar devices constructed by other groups have been used to detect blood pressure [269] and facial expressions [270].

### 5.4. Separatory and Purifying Materials

The porous structure of electrospun fibers allows not only for the adsorption of analytes but also the incorporation of materials which can catalyze degradation reactions of substances [232,294]. Furthermore, the addition of CPs in these provides a medium for transport of charges from photocatalysts like TiO_2_ [295]. It is for this reason that electrospun CPs have found application in the field of separation and purification of materials.

#### 5.4.1. Water Purification

CPs have been particularly used for water purification, especially as electrospun CPs such as PEDOT:PSS/PEO have been observed to be resistant to water degradation [232]. A nanofiber mat of PPy coated on electrospun PAN and impregnated with nonpurified multiwalled carbon nanotubes has been observed to synergistically decontaminate water. In this device, dissolved oxygen in water reacts with photoexcited electrons on PPy and other pollutants are degraded by radicals generated by charge-carrying “holes” on the carbon nanotubes [271]. In situ polymerized PEDOT on TiO_2_ nanofibers have been found to improve the photocatalytic performance of bare TiO_2_ and induce the degradation of the model pharmaceutical contaminant phenazopyridine in water [272]. In situ polymerized PANI on electrospun PS was found to be able to be de-doped and complexed by heavy-metal ions such as Hg(II), Cd(II), Pb(II), Cr(VI), and Cu(II), resulting in their remediation from water [273]. PANI/PAN membranes, manufactured through coating of in situ polymerization of PANI on electrospun PAN and electrospinning of a blend of PANI and PAN, were observed to effectively separate industrial oils from water due to the presence of an electric double layer and hydrophilic groups on PANI [233]. 

#### 5.4.2. Biopurification

The separatory properties of electrospun CPs can be applied to the isolation and potential degradation of biological contaminants. PANI-coated PU electrospun fibers were used in a microfluidic device to extract and spectrophotometrically detect morphine, codeine, and papaverine in urine [274]. PANI-coated polycaprolactam nanofibers were used to adsorb angiotensin II receptor antagonists like valsartan, losartan, and irbesartan from blood plasma via ion exchange [275]. 

During kidney failure, protein-bound uremic toxins (PBUTs) accumulate in the blood and are difficult to remove with traditional dialysis techniques [234]. Yen and coworkers used PEDOT:PSS-containing nanofibers to create a bioelectronic interface, facilitating electrical stimulation to remove PBUTs via an improved hemodialysis approach [234]. The team electrospun a mixture of multiwalled carbon nanotubes (MWCNTs), PEDOT:PSS, PEO (carrier polymer), and (3-glycidyloxypropyl)trimethoxysilane (to promote adhesion of the fibers to the substrate) directly onto conventional poly(ether sulfone) dialysis membranes. The resultant nanofiber mats exhibited excellent biocompatibility and proved very effective at removing uremic toxins.

### 5.5. Smart Devices

Electrospun CP-based nanofibers have been used in the development of “smart” devices which have tunable optical properties based on the application of a changing electric field or temperature. These devices not only have quick response times to changes in stimuli but also stability in the response after multiple cycles of loading/unloading of the stimulus. 

#### 5.5.1. Pressure- and Temperature-Dependent Applications

**Figure 24:**
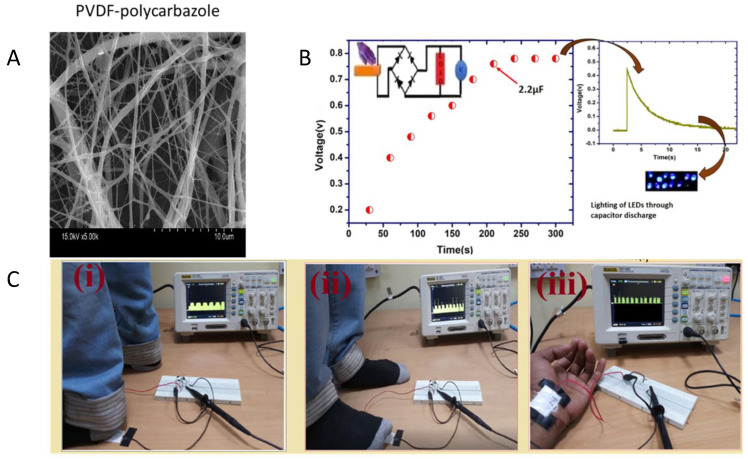
Energy-harvesting device constructed from PVDF–PCZ [235]: (**A**) SEM image of PVDF–PCZ nanofibers; (**B**) voltage measurements of the PVDF–PCZ nanocomposite showing the charging of a capacitor, and subsequent illumination of five LEDs after discharging; (**C**) types of human motion that generated voltage in the nanocomposite: (**i**) heel movement, (**ii**) toe movement, and (**iii**) wrist bending. Reprinted with permission from [235]. Copyright 2021, American Chemical Society.

Electrospun CP-based nanofibers have been used in the field of clothing, and, interestingly, in the harvesting of energy from human motion or the environment. Devices based on PEDOT and polycarbazole (PCZ) have good strain capabilities for stretchability and robustness, structural integrity, and washability with retention of properties. A PEDOT:PSS-coated composite of multiwalled carbon nanotubes/lauric acid/thermoplastic urethane has been shown to store energy and convert an applied voltage to thermal energy, and release the energy when the electric stimulus is removed [276]. The addition of PCZ to electrospun PVDF was shown to stabilize the beta phase of PVDF with the highest piezoelectric coefficient and thus enable the material to harvest energy from periodic vertical compressive motions like toe and heel motions, wrist bending, and finger movements, at a level where it is able to light up multiple light-emitting diodes (Figure 24) [235]. A composite of PEDOT:PSS/PVA coated with silver nanoparticles on a poly(ethylene terephthalate) film was demonstrated to convert heat to electricity via phonon scattering at the silver–CP interface [236]. 

**Figure 25:**
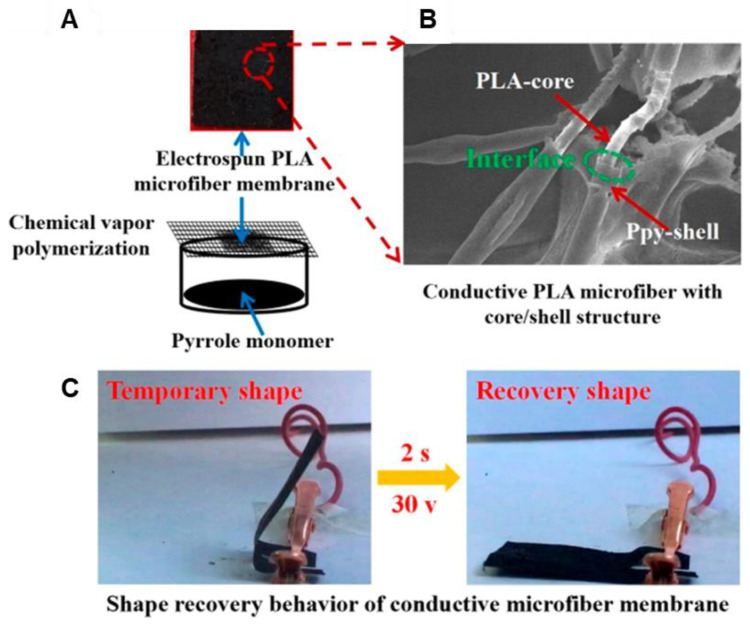
Shape memory device constructed from PPy coated on electrospun PLA [277]: (**A**) schematic of in situ polymerization of PPy onto electrospun PLA; (**B**) SEM image showing core/shell structure of nanocomposite with PLA as the core and PPy as the shell; (**C**) effect of application and removal of 30 V across composite resulting from the conduction of a heat-generating electric current. Reprinted with permission from [277]. Copyright 2018, American Chemical Society.

Devices made from electrospun CPs have been observed to quickly and uniformly heat up as a consequence of the application of voltage. The application of a voltage across a device made from PPy coated on electrospun PLA has been reported to induce quick uniform heating and resultant deformation, with the deformation retained until the applied voltage is removed and the material returns to room temperature (Figure 25) [277]. Conversely, a device has been made which changes its electrical properties with changes in temperature. A mixture of PEDOT:PSS and poly(N-isopropylacrylamide-co-N-methylol acrylamide) was electrospun into mats and was observed to change in surface resistance in response to temperature changes with a high sensitivity in the range of 20–50 °C [237].

#### 5.5.2. Optical Applications

**Figure 26:**
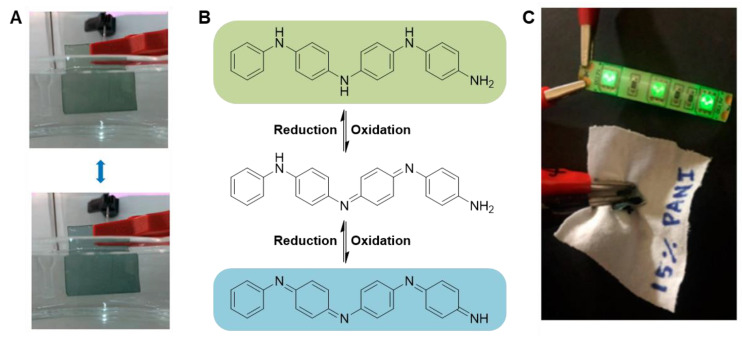
Electrochromic and conductive property exhibited by electrospun PANI/silk fibroin nanofibers [239]: (**A**) green coloration observed on PANI/silk fibroin after the application of 0.2 V and blue coloration observed after application of 0.6 V; (**B**) schematic of the structural and color changes accompanying the oxidation and reduction of PANI; (**C**) the same PANI/silk fibroin nanofibers sewn onto cotton fabric and used as a conductor to light up LEDs. Reprinted with Ref. [239], available at https://doi.org/10.3390/polym12092102, accessed on 1 December 2022.

Electrospun CP-based nanofibers have been observed to undergo color changes on the basis of their oxidation state with the uniformity, alignment, and compactness of the device. These factors have been observed to affect not only the distribution of color but also the mechanical properties of the resultant material [238]. These have potential applications in the fields of smart textiles. PANI/PVA electrospun composite nanofibers were observed to change in color from green to blue to yellow [239]. The structure of the electrochromic device constructed from these composite nanofibers is composed of a PMMA gel electrolyte, composed of LiClO_4_ and PMMA, coated onto the PANI/PVA nanofibers. The same behavior was observed in a device constructed from electrospun PANI/silk fibroin nanofibers, with the addition of good processability and conductivity sufficient to light up LEDs (Figure 26) [239]. A color change from light brown to transparent was observed as an applied voltage was increased across a device made from electrospun mixtures of PPy/WO_3_ synthesized in ionic liquids [240]. Fluorescence was also observed in devices with electrospun CPs. Blue emission was observed in an electrospun blend of a PFO-based polymer and acrylonitrile butadiene rubber, with retention of fluorescence after 200 cycles of stretching and releasing. The addition of acrylonitrile butadiene rubber served to increase flexibility and reduced aggregation [241]. 

## 6. Conclusions and Future Outlook 

Electrospinning polymers leads to high surface area and potential for alignment, giving rise to applications in textiles and filtration. The additional benefits of tunable optoelectronic properties of CPs lead to even more nanofiber applications, including sensors, tissue engineering, drug delivery, electrochromics, energy storage, and photovoltaics. Thus, even though electrospinning CPs is very challenging, researchers continue to develop approaches to overcome the barriers. 

Rigidity and low molecular weight are the two main challenges for neat electrospinning of CPs; instead of regular nanofiber formation, bead-on-a-string geometries are formed [176,191], or worse, no fiber formation occurs at all. While there are very few instances of neat CP electrospinning, several workarounds are commonly employed. Blending a CP with a carrier polymer, even at very low concentrations, can form good nanofibers with surprisingly high conductivities [198]. At higher carrier polymer concentrations, many of the desirable properties of the CP (conductivity, electroactivity) are lost. Instead, coaxial electrospinning can be used to produce core/shell nanofibers, and removal of the carrier polymer can produce neat CP nanofibers [173,182]. Alternatively, CP coatings can be deposited on electrospun commodity polymers, yielding an electroactive surface that can provide a combination of the mechanical properties of the commodity polymer with the optoelectronic properties of the CP. Coating deposition methods include solution-based chemical oxidative polymerization [242,243,244,245], vapor-phase deposition [186], and dip coating [246,247].

Overcoming the barriers of CP electrospinning has led to investigation of CP nanofibers for use in a wide variety of applications. The biocompatibility of several CPs has been demonstrated [205,254,255], leading to applications in the biomedical field, where the high surface areas of electrospun nanofibers often prove advantageous. Biomedical applications include wound healing [204,278], photothermal therapy [252], drug delivery [279], nerve regeneration [245], tissue engineering [205,206,207,254,255], and biosensors [201,228,229,230,231,262,263,264,265,266,267,268,269,270]. Electronic device applications include photovoltaics [202,206,210,257], field-effect transistors [191,198,200], and diodes [215]. 

The high surface areas are also useful for sensors, where surface area may directly relate to sensing ability [286]. Changes in conductivity are often employed as the sensing mechanism [216,217,218,219,220,221,259,288,296], while fluorescence quenching [223,260], color changes [227], pH changes [201], and mechanical deformation/pressure changes [231,267,268,269,270] have also been employed. Sensors have been developed for gases [216,217,219,220,221,222,259,296], humidity [224,225,287,288,289], explosives [218], chemical warfare agents [260,289], ions [226,227,261], and, as mentioned above, a wide variety of bioactive species such as additives, antigens, and biomolecules for detection of medical conditions. Wearable sensors have been developed to detect harmful substances, analyze bodily fluids, monitor vital signs, and record motion [201,231,266,267,268,269,270].

CP nanofibers have also been explored for use in separations and purification, because the high-surface-area, porous structures can be used to absorb species or immobilize reactants. Removal/extraction occurs via ion exchange [273], chemical degradation [232,271,272] [239,240,241], or adsorption [233,234,274,275]. Wearable smart materials can utilize interconversion of heat and electricity [235,236,237,276,277] or capitalize on dynamic optoelectronic behavior [240,241]. 

CPs, commodity polymers, and, in some cases, nanoparticles, can be combined in seemingly innumerable ways to form electrospun nanofibers with a wide range of properties. The ability to control the properties by changing the formulation is one of the reasons these materials are of such interest. Efforts will continue to directly electrospin neat CPs by increasing molecular weight and improved solubility, with the possible end goal of enhanced conductivity in aligned CP nanofibers. As the utility of these materials continues to become more widely known, additional unexpected applications will arise. When applications of CP nanofibers are further developed, an increased focus on production-scale electrospinning equipment is sure to follow. 

## Figures and Tables

**Table 1:** Advantages and disadvantages of approaches to preparation of CP nanofibers.

Approach	Advantages	Disadvantages
**Neat electrospinning**	Entire nanofiber mat is electroactive [177]	Challenging due to low chain entanglement [172]
	High conductivity possible [177]	May suffer from poor mechanical properties [178,179,180,181]
**Coaxial electrospinning**	Can remove nonconductive polymer after electrospinning [182] Nonconductive polymer provides mechanical strength [183]	Requires special coaxial needle and control of additional parameters [184]
**Co-electrospinning with carrier polymer**	Applicable to a wide range of CPs [179] Combine beneficial properties of carrier polymer and CP [175]	Lower conductivity than neat CPs [172,179,182,185]
**Post-electrospinning coating**	Applicable to a wide range of CPs Combination of substrate polymer and CP enhances mechanical strength [90,180] Can remove nonconductive polymer after electrospinning [186,187] Less CP may be needed	Requires additional step May suffer from poor adhesion [188] Bulk conductivity may be poor [179]

## Abbreviations

CP	Inherently conductive polymer
DMF	N,N-Dimethylformamide
E_g_	Bandgap
e-PU	Electrospun polyurethane
FET	Field-effect transistor
HDF	Human dermal fibroblasts
LED	Light-emitting diode
MEH-PPV	Poly[2-methoxy-5-(2-ethylhexyloxy)-1,4-phenylenevinylene]
Mv	Viscosity-average molecular weight
Mw	Weight-average molecular weight
MWCNT	Multiwalled carbon nanotubes
NBR	Nitrile butadiene rubber
OLED	Organic light-emitting diode
OTFET	Organic thin-film transistor
P3AT	Poly(3-alkylthiophene)
P3HT	Poly(3-hexylthiophene)
PA	Polyacetylene
PA-6	Polycaprolactam
PAA	Poly(acrylic acid)
PAN	Poly(acrylonitrile)
PANI	Polyaniline
PCL	Polycaprolactone
PCZ	Polycarbazole
PDBTT	Poly(N,N-bis(2-octyldodecyl)-3,6-di(thiophen-2-yl)-2,5-dihydropyrrolo [3,4-c]pyrrole-1,4-dione-alt-thieno [3,2-b]thiophene)
PE	Polyethylene
PEDOT	Poly(3,4-ethylenedioxythiophene)
PEDOT:PSS	Poly(3,4-ethylenedioxythiophene):poly(styrene sulfonate)
PEGDMA	Poly(ethylene glycol) dimethacrylate
PEO	Poly(ethylene oxide)
PFO	Polyfluorene
PindPLGA	PolyindolePoly(lactic-co-glycolic acid)
PMMA	Poly(methyl methacrylate)
PNVPY	Poly(N-vinylpyrrole)
PP	Polypropylene
PPV	Poly(phenylene vinylene)
PPy	Polypyrrole
PS	Polystyrene
PSS	Poly(styrene sulfonate)
PT	Polythiophene
PVA	Poly(vinyl alcohol)
PVAc	Poly(vinyl acetate)
PVC	Poly(vinyl chloride)
PVDF	Poly(vinylidene difluoride)
PVP	Poly(N-vinylpyrrolidone)
RH	Relative humidity
SEM	Scanning electron micrograph
TCB	1,2,4-trichlorobenzene
TEM	Tunneling electron micrograph
